# Efficacy and Safety of Belantamab Mafodotin with Bortezomib plus Dexamethasone in Patients with Relapsed/Refractory Multiple Myeloma: The DREAMM-6 Arm B Trial

**DOI:** 10.1158/1078-0432.CCR-25-3216

**Published:** 2026-03-02

**Authors:** Rakesh Popat, Bradley Augustson, Paul Cannell, Keith Stockerl-Goldstein, Andrew Spencer, Amit Khot, Ajay Nooka, Nashita Patel, Ravi S. Kasinathan, Astrid McKeown, Amy Curry, Rachel Rogers, Mehreen Shaikh, Fernando Carreno, Sumita Roy-Ghanta, Joanna Opalinska, Hang Quach

**Affiliations:** 1 National Institute for Health Research University College London Hospitals Clinical Research Facility, University College London Hospitals National Health Service Foundation Trust, London, United Kingdom.; 2 Sir Charles Gairdner Hospital and Linear Clinical Research, Perth, Australia.; 3 Department of Haematology, Fiona Stanley Hospital, Perth, Australia.; 4 Washington University School of Medicine, St. Louis, Missouri.; 5 Australian Centre for Blood Diseases, Alfred Health, Monash University, Melbourne, Australia.; 6 Department of Malignant Haematology and Stem Cell Transplantation, Alfred Hospital, Melbourne, Australia.; 7 Clinical Haematology, Peter MacCallum Cancer Centre, Royal Melbourne Hospital, University of Melbourne, Melbourne, Australia.; 8 Department of Hematology and Medical Oncology, Winship Cancer Institute of Emory University, Atlanta, Georgia.; 9 GSK, London, United Kingdom.; 10 GSK, Upper Providence, Pennsylvania.; 11 GSK, Stevenage, United Kingdom.; 12 GSK, Durham, North Carolina.; 13 University of Melbourne, St. Vincent’s Hospital Melbourne, Melbourne, Australia.

## Abstract

**Purpose:**

The phase I/II DREAMM-6 arm B study (NCT03544281) explored belantamab mafodotin combined with bortezomib/dexamethasone (BVd) in relapsed/refractory multiple myeloma (RRMM).

**Patients and Methods:**

Adults with RRMM were enrolled sequentially in two belantamab mafodotin (intravenous) dose-escalation (DE) cohorts (2.5 then 3.4 mg/kg every 3 weeks). Additional patients enrolled sequentially to eight dose-expansion cohorts: 1.9, 2.5, or 3.4 mg/kg every 3 weeks; 2.5 or 3.4 mg/kg every 3 weeks split dose (days 1 and 8); 1.9 or 2.5 mg/kg every 6 weeks; or 2.5 mg/kg in cycle 1 stepped down to 1.9 mg/kg every 6 weeks thereafter. Patients received bortezomib twice weekly and dexamethasone four times weekly. Endpoints were dose-limiting toxicities (DLT; DE), adverse events (AE), serious AEs (SAE; DE and expansion), overall response rate (ORR; expansion), and pharmacokinetics.

**Results:**

One hundred seven patients (median 4 prior lines of therapy) received BVd (*n* = 12–18/cohort). The median follow-up was 15.2 to 25.4 months. No DLTs occurred during DE. The most common grade 3/4 AE was keratopathy (53%). Protocol-defined ocular events (change in best corrected visual acuity and/or corneal examination findings) were reported in 93% of patients (grade 3/4: 77%). Twenty-eight (26%) patients experienced any study treatment-related SAEs; 3 of 7 fatal SAEs had a treatment-related primary cause. The ORR was 70% (95% confidence interval, 60.5–78.6). Higher initial exposures had higher probabilities of response and ocular events; lower exposures resulted in fewer deep responses, with small differences in ocular events.

**Conclusions:**

BVd demonstrated manageable safety and clinical activity across all dosing cohorts in heavily pretreated RRMM, supporting the 2.5 mg/kg every 3 weeks dose.

**Translational Relevance**

By evaluating eight different dosing regimens of belantamab mafodotin plus bortezomib and dexamethasone (BVd), including 2.5 mg/kg every 3 weeks, as used in the phase III DREAMM-7 study, this study demonstrated that BVd has a manageable safety profile and is highly efficacious in a relapsed/refractory multiple myeloma (RRMM) population who were heavily pretreated (median 4 prior lines). Exposure–response relationships demonstrated that higher exposures lead to greater likelihood of response but also more ocular adverse reactions, whereas lower exposures resulted in fewer deep responses, with small differences in ocular events. Patient-reported pain, reported in terms of frequency, severity, and interference, was similar across all cohorts. The results support previously published phase III DREAMM-7 trial data for belantamab mafodotin in combination with Vd in the treatment of RRMM and may help inform clinical practice.

## Introduction

Multiple myeloma is the second most common hematologic malignancy, is generally considered incurable, and is characterized by the infiltration of malignant plasma cells in the bone marrow (1). Treatment of multiple myeloma has advanced with the development of novel therapies and regimens, including triplet and quadruplet combinations; however, most patients relapse or become refractory to treatment, requiring multiple lines of therapy over time (2, 3). Over the disease course, periods between relapses become shorter and empiric decisions around the optimal use of the available therapeutic options become more challenging, highlighting the need for new, effective treatments for relapsed/refractory multiple myeloma (RRMM; ref. 4).

B-cell maturation antigen (BCMA) is an important and novel target in the treatment of RRMM because of its high level of expression on multiple myeloma plasma cells and critical role in plasma cell survival and proliferation (5). Belantamab mafodotin is a first-in-class antibody–drug conjugate (ADC) consisting of a humanized, afucosylated monoclonal antibody (mAb) which targets BCMA and is conjugated to the microtubule inhibitor monomethyl auristatin F (MMAF; ref. 6). Belantamab mafodotin has multiple mechanisms of action, including antibody-dependent cellular cytotoxicity, antibody-dependent cellular phagocytosis, and immunogenic cell death (6, 7). Preclinical studies in cell lines and *in vivo* xenograft models have shown that belantamab mafodotin is synergistic with standard-of-care agents, such as bortezomib, lenalidomide, pomalidomide, and dexamethasone, to enhance antitumor activity, providing a rationale for clinical studies into these combination regimens (6, 8).

Belantamab mafodotin has shown clinical benefit in combination regimens for RRMM (9, 10). Recent findings from the phase III DREAMM-7 study (NCT04246047) showed that in patients with RRMM who had received ≥1 prior line of treatment (LOT), belantamab mafodotin plus bortezomib and dexamethasone (BVd) demonstrated a progression-free survival (PFS) benefit of 36.6 months versus 13.4 months (9), which was maintained following the subsequent line of therapy (PFS2) with a hazard ratio (HR) of 0.56 [95% confidence interval (CI), 0.41–0.76; ref. 11]; there was also an overall survival (OS) benefit with a statistically significant 42% reduction in the risk of death compared with daratumumab plus bortezomib and dexamethasone (DVd; HR: 0.58; 95% CI, 0.43–0.79; *P* = 0.00023) after a median follow-up of 39.4 months (12). DREAMM-7 was a phase III, randomized, open-label study designed to evaluate the safety and efficacy of BVd versus DVd in participants with relapsed recurrent multiple myeloma. The primary endpoint of PFS was significant for BVd as was the OS secondary endpoint (11, 13).

DREAMM-6 (NCT03544281) is a phase I/II open-label dose-escalation (DE)/dose-expansion study that aimed to investigate the safety and clinical activity of belantamab mafodotin at various doses and schedules in combination with standard therapies. In arm A of DREAMM-6 (reported elsewhere), belantamab mafodotin was combined with lenalidomide and dexamethasone and demonstrated an overall response rate (ORR) of 66.7% (95% CI, 51–80) across dose cohorts, with a manageable safety profile in patients who had received a median (range) of 3 (1–10) prior LOTs (14). Here, we report DREAMM-6 Arm B findings, which investigated BVd across multiple dosing cohorts and aimed to identify the optimal dose of belantamab mafodotin when used in this combination.

## Patients and Methods

### Study design and patients

Arm B consisted of an abbreviated DE phase to evaluate the safety and tolerability of BVd with escalating doses of belantamab mafodotin and a dose-expansion phase to further evaluate select doses and dosing schedules for safety and clinical activity (Supplementary Fig. S1). In the DE phase, eligible patients assigned to arm B, at the discretion of the investigator, initially received intravenous belantamab mafodotin 2.5 mg/kg as a single dose on day 1 every 3 weeks, and if safety/tolerability were confirmed, the dose was escalated to 3.4 mg/kg on day 1 every 3 weeks in the next group of patients. A maximum of six participants were assigned to each dose during the DE phase, with the total number of patients in each cohort being informed by dose-limiting toxicities (DLT) that occurred. If the 2.5 mg/kg dose was not tolerated, an additional lower dose (1.9 mg/kg) was to be evaluated. The 1.9 and 2.5 mg/kg every 3 weeks starting doses were selected for their potential to provide benefit to patients while also allowing careful assessment of safety (15, 16). For DE and dose expansion, bortezomib 1.3 mg/m^2^ was administered by subcutaneous or intravenous infusion on days 1, 4, 8, and 11, and dexamethasone 20 mg was administered orally or by intravenous infusion on days 1, 2, 4, 5, 8, 9, 11, and 12 of each 21-day cycle, up to eight cycles. Full details on the bortezomib and dexamethasone dosing schedules are provided in Supplementary Methods S1.

In the dose-expansion phase, patients were assigned sequentially in blocks of 3 patients to 1 of 8 belantamab mafodotin dosing cohorts until the cohorts that opened later contained approximately 12 patients. Belantamab mafodotin was administered intravenously in the following cohorts:

- Every 3 weeks dosing on day 1 of each cycle:
  - 1.9 mg/kg.
  - 2.5 mg/kg.
  - 3.4 mg/kg.
- Every 3 weeks split: doses administered as a 50:50 split dose on days 1 and 8 of each cycle:
  • 2.5 mg/kg (1.25 mg/kg on days 1 and 8).
  • 3.4 mg/kg (1.7 mg/kg on days 1 and 8).
- Every 6 weeks on day 1 of each cycle:
  • 1.9 mg/kg.
  • 2.5 mg/kg.
- Step-down (S/D):
  • 2.5 mg/kg on day 1 of cycle 1, followed by 1.9 mg/kg every 6 weeks thereafter (2.5–1.9 mg/kg S/D every 6 weeks).

Split-dose regimens (2 equal doses 1 week apart) were evaluated to determine if there was an improvement of the benefit/risk profile due to a reduction in the maximum concentration while maintaining similar exposure over a cycle to the once every 3 weeks dosing. Belantamab mafodotin exposure is expected to increase over time due to reduced clearance and its long half-life (17); therefore, every 6 weeks dosing, representing 3 to 4 half-lives, was selected to reduce belantamab mafodotin accumulation over time, with 2.5 to 1.9 mg/kg S/D every 6 weeks dosing providing an alternative to 2.5 mg/kg every 6 weeks dosing.

For both phases, dose modifications (dose interruptions and reductions) for all study drugs were permitted after the completion of cycle 1 to manage tolerability and adverse events (AE). As belantamab mafodotin 1.9 mg/kg was the lowest dose in the study, reductions for this dose were not permitted in select cohorts (1.9 mg/kg every 3 weeks, 1.9 mg/kg every 6 weeks, and 2.5–1.9 mg/kg S/D every 6 weeks). Patients were treated with BVd up to cycle 8 and then belantamab mafodotin alone until confirmed progressive disease, death, start of a new anticancer treatment, withdrawal of consent, or end of the study, whichever occurred first.

Eligible patients were ≥18 years of age, with a confirmed diagnosis of multiple myeloma per International Myeloma Working Group (IMWG) criteria (18), measurable disease, Eastern Cooperative Oncology Group performance status of 0 to 2, and ≥1 prior LOT (prior bortezomib exposure was allowed) with documented disease progression during or after their most recent therapy (full patient eligibility criteria are listed in Supplementary Table S1).

This study was conducted in accordance with the Declaration of Helsinki and Council for International Organizations of Medical Sciences International Ethical Guidelines, applicable Good Clinical Practice guidelines, and applicable local laws and regulations. The study protocol was reviewed and approved by institutional review boards or an independent ethics committee (detailed in the Supplementary Methods S1). Patients provided signed informed consent.

### Endpoints and assessments

For DE, the primary endpoints were the number and proportion of patients with a DLT and AEs, including serious AEs (SAE). DLTs were assessed in the first 21-day cycle; an event was considered a DLT if it was attributed to belantamab mafodotin and met one of the DLT criteria described in Supplementary Methods S1 and Supplementary Table S2. For dose expansion, primary endpoints were AEs, SAEs, and ORR. The definition of ORR was the proportion of patients achieving a confirmed partial response (PR) or better according to IMWG response criteria (18), assessed every 3 weeks (±3 days) by the investigator, in which patients with unknown or missing responses were treated as nonresponders. All AEs were collected from the start of treatment until at least 70 days following discontinuation of study treatment; belantamab mafodotin treatment could continue if bortezomib or dexamethasone was discontinued. AEs were graded according to National Cancer Institute Common Terminology Criteria for Adverse Events (NCI-CTCAE) version 4.03 (19).

Secondary endpoints for both phases included pharmacokinetic (PK) parameters during cycle 1, incidence of anti-drug antibodies (ADA) against belantamab mafodotin predose in cycle 1 and at selected subsequent cycles, health-related quality of life (HRQoL) assessments, and AEs of special interest (AESI). PK parameters for belantamab mafodotin, total mAbs, and cys-mcMMAF were derived using noncompartmental analysis. Blood samples for PK were taken across multiple days during cycle 1, selected subsequent cycles, and at the last treatment visit; samples for bortezomib PK were collected before and after dose for cycle 1, day 1 for every 3 weeks cohorts only. Further details on the PK methodology can be found in Supplementary Methods S1. At a minimum, samples for serum BCMA (sBCMA) were collected at the same timepoints as PK samples. Exposure–efficacy and exposure–safety analyses were also carried out to assess the relationship between belantamab mafodotin exposure with clinical activity and AEs and were performed using population PK analysis (see Supplementary Methods S1 and Supplementary Table S3 for further details). HRQoL assessments included maximum post-baseline scores of the Patient-Reported Outcomes version of the CTCAE (PRO-CTCAE), change from baseline in Ocular Surface Disease Index (OSDI) score, and National Eye Institute Visual Function Questionnaire (NEI-VFQ) 25 score (20, 21). AESIs included ocular adverse reactions (oAR), ocular events, thrombocytopenia, and infusion-related reactions. Analysis of oARs included event frequency, severity, time to first event, event type, and resolution of event (by final analysis and at last visit) and were graded using the NCI-CTCAE version 4.03. Ocular events, which comprise corneal examination findings per slit-lamp examination and visual acuity changes per best corrected visual acuity (BCVA) score, were graded by ophthalmologists using the sponsor’s protocol-defined scale (Supplementary Table S4).

Exploratory efficacy outcomes, reported across DE and dose expansion, included complete response or better (≥CR) and very good PR or better (≥VGPR), time to response (TTR), time to best response, duration of response (DoR), time to progression, PFS, OS, relationship between clinical response and sBCMA levels, and minimal residual disease (MRD) negativity for patients with ≥VGPR assessed by next-generation sequencing, with a sensitivity of 10^−5^. For the all-treated population, resolution of protocol-defined grade ≥2 ocular events, ORR, ≥CR, and median PFS outcomes combining all cohorts were assessed *post hoc*.

### Statistical analysis

During DE, an alternative version of the modified toxicity probability interval design (22) was utilized to guide DE/de-escalation decisions (see Supplementary Methods S1), in which the total number of patients enrolled into the DE phase was dependent on the number of patients needed to characterize each individual cohort. Based on assumptions that three different dose levels were evaluated, it was anticipated that a maximum of six patients will be assigned to each dose. For dose expansion, up to three doses and up to four dosing schedules were evaluated with up to 12 patients enrolled at each dosing schedule, totaling approximately 96 patients. With the exception of DLT, all analyses included data pooled from patients in the DE and dose-expansion phases. The primary efficacy endpoint and all safety analyses were performed on patients who received ≥1 dose of any study treatment (all-treated population).

All safety, efficacy, and HRQoL data were reported using descriptive statistics, with median and range used for continuous variables and frequencies and percentages for categorical variables. No formal statistical hypotheses were tested. DoR, PFS, and OS outcomes were summarized using the Kaplan–Meier method with associated 95% CI estimated using the Brookmeyer–Crowley method. PK analyses were performed with data from patients in the all-treated population from whom ≥1 PK sample was obtained, analyzed, and measurable; parameters were summarized for each analyte and dosing regimen by visit using (geometric) mean and standard deviation (SD), median and range, percentage coefficient of variation (%CV), and 95% CI of log-transformed parameters. Statistical considerations for population PK, exposure–efficacy, and exposure–safety analyses are described in Supplementary Methods S1.

## Results

### Patient population

Arm B of the DREAMM-6 study was initiated on September 20, 2018, and included 26 centers across five countries (Australia, Canada, Spain, the United Kingdom, and the United States). As of the data cutoff date for the final analysis (February 28, 2023), 107 patients were enrolled and treated; there were 12 patients in each cohort except for the 2.5 mg/kg split every 3 weeks (*n* = 13), 2.5 mg/kg every 3 weeks (*n* = 18), and 3.4 mg/kg every 3 weeks (*n* = 16) cohorts. Patient status and reasons for study withdrawal are presented in Supplementary Fig. S2. Study discontinuations for any reason ranged from 50% to 83% of patients in each cohort (*n* = 69 overall); 17 patients had ongoing treatment at the time of the last patient’s final visit, and 15 of these proceeded to post-analysis continued treatment. The representativeness of this study to the overall multiple myeloma population is shown in Supplementary Table S5.

Table 1 and Supplementary Table S6 summarize baseline characteristics for each dose cohort and for the overall study population. Demographic and disease characteristics were generally similar across all cohorts. Overall, patients were predominantly White/Caucasian/European (*n* = 88, 82%), with a median (range) age of 66 (32–83) years, and 18% of patients were ≥75 years of age. Overall, 85% of patients had an International Staging System stage of I or II, 21% had extramedullary disease, and 66% had lytic bone lesions with some variation across the dose cohorts. Patients received a median (range) of 4 (1–13) prior LOTs, 90% had prior bortezomib exposure, 80% had prior lenalidomide exposure, and 45% had prior daratumumab exposure, all of which were considered similar between cohorts. The median duration of follow-up ranged from 15.2 (1.9 mg/kg every 6 weeks) to 25.4 (3.4 mg/kg every 3 weeks) months across cohorts.

**Table 1.** Patient demographics and clinical characteristics.

Parameter	1.9 mg/kg Q6W (*n* = 12)	1.9 mg/kg Q3W (*n* = 12)	2.5–1.9 mg/kg S/D Q6W (*n* = 12)	2.5 mg/kg Q6W (*n* = 12)	2.5 mg/kg split Q3W (*n* = 13)	2.5 mg/kg Q3W (*n* = 18)	3.4 mg/kg split Q3W (*n* = 12)	3.4 mg/kg Q3W (*n* = 16)	All treated (*N* = 107)
Duration of follow-up, months, median (range)^a^	15.21 (0.8–19.8)	16.15 (2.1–21.2)	17.33 (5.6–21.7)	22.32 (5.8–25.6)	16.76 (0.9–41.5)	19.50 (5.3–49.2)	19.63 (0.9–41.4)	25.41 (3.3–49.2)	17.38 (0.8–49.2)
Age, years, median (range)	63 (47–82)	66.5 (57–78)	67.5 (48–77)	66 (58–79)	66 (32–78)	67 (47–83)	64.5 (50–76)	63 (46–78)	66 (32–83)
≥75, *n* (%)	3 (25)	1 (8)	1 (8)	4 (33)	3 (23)	4 (22)	1 (8)	2 (13)	19 (18)
Male, *n* (%)	7 (58)	9 (75)	7 (58)	5 (42)	11 (85)	11 (61)	8 (67)	11 (69)	69 (64)
Race, *n* (%)	​	​	​	​	​	​	​	​	​
White/Caucasian/European heritage	11 (92)	11 (92)	10 (83)	7 (58)	11 (85)	12 (67)	11 (92)	15 (94)	88 (82)
African American/African heritage	0 (0)	0 (0)	1 (8)	3 (25)	0 (0)	4 (22)	0 (0)	1 (6)	9 (8)
Prior LOT, median (range)	3.5 (2–7)	3.5 (1–8)	4.5 (1–8)	3.5 (1–8)	4 (1–8)	3 (1–11)	3.5 (1–13)	3 (1–10)	4 (1–13)
Prior stem cell transplant, *n* (%)	11 (92)	9 (75)	10 (83)	9 (75)	9 (69)	14 (78)	12 (100)	15 (94)	89 (83)
ISS stage, *n* (%)	​	​	​	​	​	​	​	​	​
I	6 (50)	7 (58)	10 (83)	8 (67)	3 (23)	6 (33)	3 (25)	10 (63)	53 (50)
II	4 (33)	4 (33)	1 (8)	3 (25)	6 (46)	9 (50)	5 (42)	5 (31)	37 (35)
III	2 (17)	1 (8)	1 (8)	1 (8)	4 (31)	3 (17)	4 (33)	1 (6)	17 (16)

Percentages may not equate to 100 due to rounding.

Abbreviations: ISS, International Staging System; LOT, line of therapy; Q3W, every 3 weeks; Q6W, every 6 weeks; S/D, step-down.

a Defined as the time from first dose to last contact or death.

### Treatment exposure

The median duration of treatment with belantamab mafodotin (range) was between 2.1 (0.9–39.7; 2.5 mg/kg split every 3 weeks) and 11.3 (0.7–22.3; 2.5–1.9 mg/kg S/D every 6 weeks) months (Table 2). Relative dose intensity for belantamab mafodotin ranged from 47.6% (2.5 mg/kg every 3 weeks) to 70% (2.5–1.9 mg/kg S/D every 6 weeks) when measured from dose 2 to the end of treatment. Dose reductions for any therapy were reported in all cohorts, in which dose reductions were permitted, and were most commonly due to an AE, with the highest number of belantamab mafodotin dose reductions reported for the 2.5 mg/kg every 3 weeks cohort, 14 (93%) of which were due to AEs (Supplementary Table S7). Dose delays were reported in ≤50% of patients in each cohort (Supplementary Table S7). A total of 38 (35.5%) patients had any AE that led to discontinuation of any component of the study treatment (belantamab mafodotin treatment could continue if bortezomib or dexamethasone was discontinued, Table 3), and 18 (17%) patients had AEs related to belantamab mafodotin that resulted in discontinuation of any study treatment. The most common AEs leading to discontinuation of any treatment component were peripheral neuropathy (*n* = 9; 8.4%), increased alanine aminotransferase (*n* = 4; 3.7%), and keratopathy (*n* = 3; 2.8%).

**Table 2.** Treatment exposure.

Treatment exposure	1.9 mg/kg Q6W (*n* = 12)	1.9 mg/kg Q3W (*n* = 12)	2.5–1.9 mg/kg S/D Q6W (*n* = 12)	2.5 mg/kg Q6W (*n* = 12)	2.5 mg/kg split Q3W (*n* = 13)	2.5 mg/kg Q3W (*n* = 18)	3.4 mg/kg split Q3W (*n* = 12)	3.4 mg/kg Q3W (*n* = 16)	All treated (*N* = 107)
Belantamab mafodotin	​	​	​	​	​	​	​	​	​
Treatment duration^a^, months, median (range)	5.6 (0.7–19.1)	10.9 (1.4–21.8)	11.3 (0.7–22.3)	8.1 (0.7–21.9)	2.1 (0.9–39.7)	8.8 (2.1–39.7)	2.2 (0.9–34.5)	4.1 (0.7–31.1)	n/c
Dose intensity, mg/kg/interval, median (range)	1.6 (0.7–1.9)	0.9 (0.6–1.9)	1.5 (0.9–2.5)	1.6 (0.8–2.5)	2.1 (0.5–2.5)	1.1 (0.4–2.5)	2.6 (0.6–3.4)	1.6 (0.6–3.4)	n/c
Relative dose intensity^b^ %	​	​	​	​	​	​	​	​	​
Dose 1	100	100	100	100	107.7^c^	100	97.8	100	n/c
Post-dose 1	62.9	60.2	70	63.2	57.2	47.6	58.5	61.5	n/c
Bortezomib									
Treatment duration^a^, months, median (range)	4.1 (1–6.9)	5.8 (1.6–8.1)	5.7 (1–7.1)	5.8 (2.3–7.2)	3.1 (1–5.9)	5.8 (0.7–9.4)	3.1 (1–6.1)	4.1 (1.4–6)	n/c
Relative dose intensity^b^, %	68.5	61	80.7	68.4	79.3	65.7	73	66	n/c

Abbreviations: n/c, not computed; Q3W, every 3 weeks; Q6W, every 6 weeks; S/D, step-down.

a The time on study treatment did not exclude dose interruptions.

b Relative dose intensities were defined as the mean overall dose intensity for the specified dose, divided by planned dose intensity for that dose, multiplied by 100.

c One patient received 2.5 mg/kg on days 1 and 8.

**Table 3.** Safety summary.

Any AE, *n* (%)	1.9 mg/kg Q6W (*n* = 12)	1.9 mg/kg Q3W (*n* = 12)	2.5–1.9 mg/kg S/D Q6W (*n* = 12)	2.5 mg/kg Q6W (*n* = 12)	2.5 mg/kg split Q3W (*n* = 13)	2.5 mg/kg Q3W (*n* = 18)	3.4 mg/kg split Q3W (*n* = 12)	3.4 mg/kg Q3W (*n* = 16)	All treated (*N* = 107)
Related to any study treatment	12 (100)	12 (100)	12 (100)	12 (100)	12 (92)	18 (100)	12 (100)	16 (100)	106 (99)
Leading to permanent discontinuation of any study treatment	3 (25)	1 (8)	5 (42)	4 (33)	4 (31)	7 (39)	7 (58)	7 (44)	38 (36)
Belantamab mafodotin–related and leading to permanent discontinuation of any study treatment^a^	1 (8)	1 (8)	2 (17)	3 (25)	2 (15)	3 (17)	3 (25)	3 (19)	18 (17)
Leading to dose interruption/delay	11 (92)	12 (100)	12 (100)	12 (100)	12 (92)	18 (100)	11 (92)	16 (100)	104 (97)
Grade 3 or 4	12 (100)	12 (100)	11 (92)	12 (100)	13 (100)	18 (100)	12 (100)	16 (100)	106 (99)
Related to any study treatment	12 (100)	12 (100)	11 (92)	12 (100)	11 (85)	18 (100)	11 (92)	15 (94)	102 (95)
≥20% incidence^b^	​	​	​	​	​	​	​	​	​
Keratopathy^c^	4 (33)	8 (67)	8 (67)	6 (50)	7 (54)	9 (50)	4 (33)	11 (69)	57 (53)
Thrombocytopenia	4 (33)	5 (42)	3 (25)	5 (42)	10 (77)	5 (28)	8 (67)	8 (50)	48 (45)
Platelet count decreased	5 (42)	8 (67)	6 (50)	4 (33)	1 (8)	8 (44)	3 (25)	4 (25)	39 (36)

Abbreviations: AE, adverse event; MedDRA, Medical Dictionary for Regulatory Activities; Q3W, every 3 weeks; Q6W, every 6 weeks; S/D, step-down.

a Reasons for belantamab mafodotin–related discontinuations were alanine aminotransferase increase, *n* = 3; keratopathy, *n* = 3; diarrhea, *n* = 2; COVID-19 pneumonia, *n* = 2; thrombocytopenia, *n* = 1; aspartate aminotransferase increased, *n* = 1; urine albumin/creatinine ratio increased, *n* = 1; proteinuria, *n* = 1; fatigue, *n* = 1; weight loss, *n* = 1; supraventricular tachycardia, *n* = 1; and hepatic failure.

b ≥20% incidence for the all-treated population.

c MedDRA Preferred Term.

### Safety

In the DE phase, no DLTs were reported during cycle 1. Safety findings are summarized in Table 3 and Supplementary Table S8. Grade 3 or 4 AEs of any cause occurred in >90% of patients in each cohort, with the most common being keratopathy (changes to the corneal epithelium which may or may not result in changes in vision, *n* = 57, 53%), thrombocytopenia (*n* = 48, 45%), and decreased platelet count (*n* = 39, 36%). AESIs of platelet count decrease or thrombocytopenia were reported in >70% of patients in every cohort. Infusion-related reactions were infrequent across all cohorts [range: 0 (0%)–3 (25%) patients].

The most common any grade oARs [99 (93%)] were keratopathy (*n* = 94; 88%), blurred vision (*n* = 41; 38%), and reduced visual acuity (*n* = 26; 24%). In total, 98 (92%) patients experienced any grade oAR related to belantamab mafodotin. Grade 3 oARs were reported in 60 (56%) patients, whereas grade 4 oARs were reported in 2 (2%) patients. When measured on the protocol-defined scale comprising corneal examination findings and changes in BCVA detected in an ophthalmic examination, there were 168 grade ≥2 ocular events between 96 patients [median (range) duration: 120 (8–1,012) days], of which 105 events had resolved to grade 1 or below by data cutoff (Supplementary Table S8). Among 91 patients who had ongoing grade ≥2 ocular events after the end of treatment exposure, these events resolved in 31 patients and were ongoing at the final follow-up for 60 patients. The median time to onset to first grade ≥2 ocular event was between 21.5 (range, 17–87; 2.5–1.9 mg/kg S/D every 6 weeks) and 42 (range, 22–86; 2.5 mg/kg split every 3 weeks) days. The incidence of grade 3 or 4 ocular events (protocol-defined scale; Supplementary Methods S1) ranged from 58% for both 1.9 mg/kg every 6 weeks (*n* = 7) and 3.4 mg/kg split every 3 weeks (*n* = 7) to 94% (*n* = 15) for 3.4 mg/kg every 3 weeks.

A total of 63 (59%) patients had SAEs and 28 (26%) had SAEs related to any study treatment. Three fatal SAEs in which the primary causes of death were considered related to study treatment were reported: one patient in 2.5 to 1.9 mg/kg S/D every 6 weeks, COVID-19 pneumonia; one patient in 2.5 mg/kg every 6 weeks, COVID-19 pneumonia; and one patient in 3.4 mg/kg every 3 weeks, hepatic failure. In another patient in 3.4 mg/kg split every 3 weeks, disease progression was considered to be the primary cause of death, with sepsis considered related to belantamab mafodotin, bortezomib, and dexamethasone as a secondary cause of death. A summary of COVID-19 infections is shown in Supplementary Table S9. By system organ class, infections/infestations (any grade) occurred in 42% to 89% of patients across the cohorts.

### Efficacy

**Figure 1.**
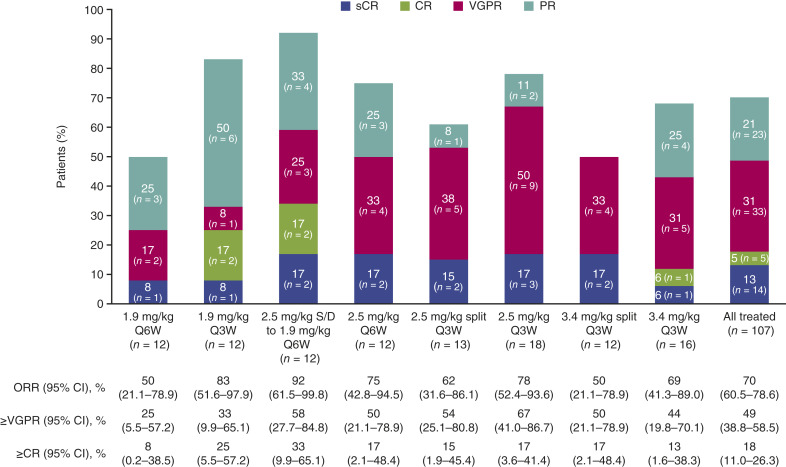
ORR in all cohorts in arm B. ORR was defined as a confirmed PR or better, and ≥CR was defined as a confirmed CR or better; patients with unknown or missing responses were treated as nonresponders. Deep responses are indicated as ≥VGPR. CI, confidence interval; CR, complete response; ORR, overall response rate; PR, partial response; Q3W, every 3 weeks; Q6W, every 6 weeks; S/D, step-down; s, stringent; VGPR, very good partial response.

The ORR ranged from 50% to 92% across cohorts and was numerically highest in the 2.5 to 1.9 mg/kg S/D every 6 weeks cohort (Fig. 1). Patients who had ≥CR ranged from 8% (*n* = 1; 1.9 mg/kg every 6 weeks) to 33% (*n* = 4; 2.5–1.9 mg/kg S/D every 6 weeks). Patients who had ≥VGPR ranged from 25% (*n* = 3; 1.9 mg/kg every 6 weeks) to 67% (*n* = 12; 2.5 mg/kg every 3 weeks). The median TTR ranged from 0.7 to 1.4 months (Table 4). The median DoR was reached in 4 of 8 cohorts and ranged from 8.7 to 29.7 months. The median PFS at the time of the final analyses ranged from 6.3 months in the 1.9 mg/kg every 6 weeks cohort to 24.2 months in the 3.4 mg/kg split every 3 weeks cohort. The median OS was reached in 3 of the 8 cohorts and ranged from 19.7 months in the 2.5 to 1.9 mg/kg S/D every 6 weeks cohort to 30.4 months in the 2.5 mg/kg every 3 weeks cohort. Of 26 patients with ≥VGPR who had MRD data, 13 achieved MRD-negative status which ranged from 0% to 17% across cohorts (Supplementary Table S10).

**Table 4.** TTR, time to best response, DoR, PFS, and OS.

​	1.9 mg/kg Q6W (*n* = 12)	1.9 mg/kg Q3W (*n* = 12)	2.5–1.9 mg/kg S/D Q6W (*n* = 12)	2.5 mg/kg Q6W (*n* = 12)	2.5 mg/kg split Q3W (*n* = 13)	2.5 mg/kg Q3W (*n* = 18)	3.4 mg/kg split Q3W (*n* = 12)	3.4 mg/kg Q3W (*n* = 16)	All treated (*N* = 107)
Duration of follow-up, months, median (range)	15.2 (0.8–19.8)	16.2 (2.1–21.2)	17.3 (5.6–21.7)	22.3 (5.8–25.6)	16.8 (0.9–41.5)	19.5 (5.3–49.2)	19.6 (0.9–41.4)	25.4 (3.3–49.2)	17.4 (0.8–49.2)
​	*n* = 6	*n* = 10	*n* = 11	*n* = 9	*n* = 8	*n* = 14	*n* = 6	*n* = 11	​
TTR, months, median (95% CI)	1.4 (1.3–2.8)	1.4 (0.7–2.4)	1.4 (1–1.6)	1.4 (0.7–1.6)	0.7 (0.7–1.5)	0.7 (0.7–1.4)	0.8 (0.7–1.8)	0.7 (0.7–1.5)	n/c
Time to best response, months, median (95% CI)	1.5 (1.4–8.9)	3.6 (1.4–6.1)	2.8 (1.4–8.9)	2.8 (0.7–6.2)	1.7 (0.7–4.1)	2.8 (0.7–5.9)	2.5 (0.7–13.4)	3.5 (0.7–8.3)	n/c
Time to progression, months, median (95% CI)	6.7 (0.7–12.1)	13.2 (1.4–NE)	16 (6–NE)	9.8 (2.1–21.2)	12.7 (0.7–NE)	10.8 (8.3–NE)	24.2 (0.8–NE)	20.1 (2.9–32.5)	n/c
DoR, *n*	6	9	11	9	8	14	6	11	74
Disease progression or death due to disease progression, *n* (%)	4 (67)	3 (33)	4 (36)	6 (67)	3 (38)	5 (36)	2 (33)	6 (55)	33 (45)
Estimate, months, median (95% CI)	8.7 (4.4–NE)	NR (2.9–NE)	NR (4.4–NE)	9.7 (2.8–19.1)	20.3 (3.5–NE)	NR (7.6–NE)	NR (5.5–NE)	29.7 (4.9–31.8)	19.1 (10.9–31.8)
PFS	​	​	​	​	​	​	​	​	​
Disease progression or death, *n* (%)	10 (83)	5 (42)	6 (50)	10 (83)	7 (54)	10 (56)	6 (50)	11 (69)	65 (61)
Estimate, months, median (95% CI)	6.3 (0.7–12.1)	13.2 (1.4–NE)	16 (5.6–NE)	9.2 (2.1–16.8)	12.7 (0.7–NE)	10.8 (8.3–NE)	24.2 (0.8–NE)	14.1 (2.9–32.5)	10.8 (8.3–16)
OS	​	​	​	​	​	​	​	​	​
Death, *n* (%)	4 (33)	2 (17)	4 (33)	5 (42)	5 (38)	8 (44)	6 (50)	4 (25)	38 (36)
Estimate, months, median (95% CI)	NR (1.1–NE)	NR (13.6–NE)	19.7 (8.5–NE)	NR (8.3–NE)	NR (1.3–NE)	30.4 (17.1–NE)	25.9 (3.6–NE)	NR (12.6–NE)	NR (25.9–NE)

CIs were estimated using the Brookmeyer–Crowley method.

Abbreviations: CI, confidence interval; DoR, duration of response; n/c, not calculated; NE, not estimable; NR, not reached; OS, overall survival; PFS, progression-free survival; Q3W, every 3 weeks; Q6W, every 6 weeks; S/D, step-down; TTR, time to response.

### PK, exposure–response, sBCMA, and ADA

**Figure 2.**
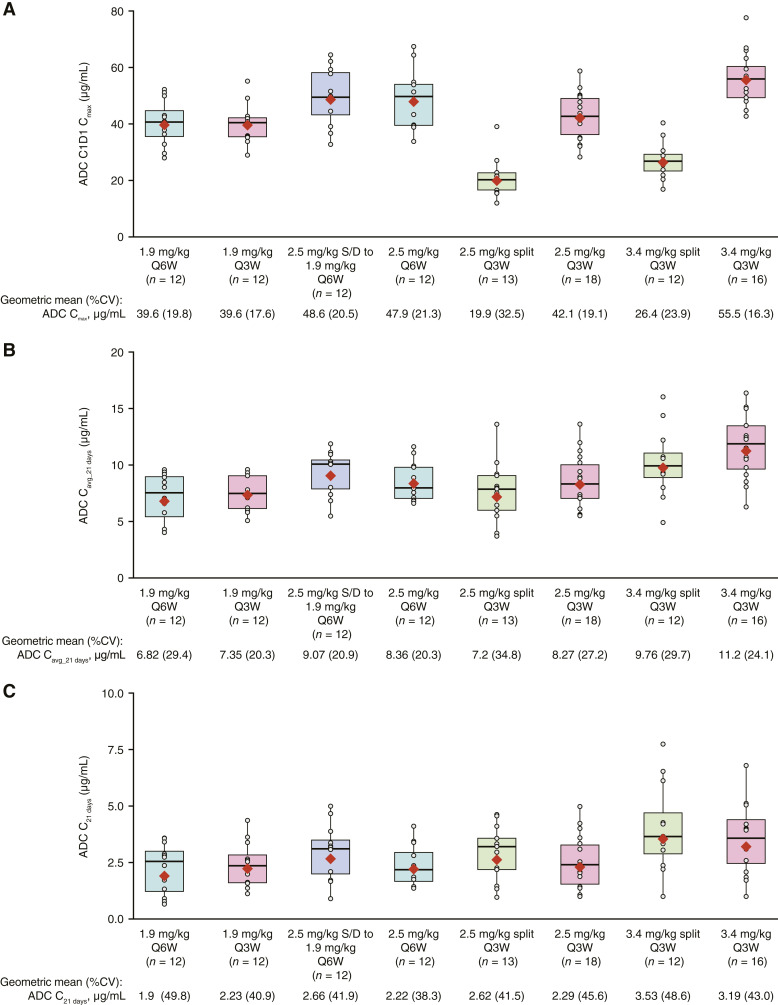
Belantamab mafodotin concentrations in arm B cohorts. Peak belantamab mafodotin concentrations during cycle 1 on day 1 (**A**), average belantamab mafodotin concentrations over cycle 1 (**B**), and belantamab mafodotin concentration at the end of cycle 1 (**C)**; exposures derived from population PK analysis. Boxplots show the distribution of the exposure parameter. The lower and upper hinges correspond to the first and third quartiles. The horizontal line inside each box represents the median. The whiskers extend from the hinge to ±1.5 × interquartile range (IQR). The gray circles and red diamond represent each individual exposure parameter and the geometric mean, respectively. %CV was calculated as 100 × SQRT [exp(SD^2^) − 1], where SD was used on a log scale. ADC, antibody-drug conjugate; C1D1, cycle 1, day 1; C_max_, maximum concentration; C_avg_21 days_, average concentration over 21 days; C_21 days_, concentration at 21 days; CV, coefficient of variation; SD, standard deviation; SQRT, square root.

Noncompartmental PK parameter values for belantamab mafodotin (ADC) at cycle 1 are presented in Supplementary Table S11. PK analysis for subcutaneous bortezomib was performed for every 3 weeks cohorts only; geometric mean of maximum concentration (C_max_; %CV) on day 1 of cycle 1 ranged from 15.88 ng/mL [42.1% (*n* = 15); 3.4 mg/kg cohort] to 14.45 ng/mL [50.2% (*n* = 11); 1.9 mg/kg cohort]. As intravenous bortezomib was only received by two patients, PK data for the intravenous formulation are not reported here. Population PK analysis showed that the belantamab mafodotin monotherapy PK model adequately described the ADC PK profiles of patients receiving BVd (23). In PK analyses of cycle 1 on day 1, belantamab mafodotin C_max_ values were lowest for the 2.5 mg/kg split every 3 weeks cohort (Fig. 2A). Average belantamab mafodotin concentrations over the 21 days in cycle 1 (C_avg_21 days_) and concentration at the end of cycle 1 (ADC C_21 days_) largely overlapped among cohorts, with a trend suggesting a modest increase in exposure for the 2.5 and 3.4 mg/kg cohorts, within the variability of the data (Fig. 2B and C). Belantamab mafodotin C_avg_21 days_ showed an inverse association with baseline sBCMA, IgG, and β2-microglobulin levels and a positive correlation with baseline albumin levels.

Exposure–efficacy analyses showed that a greater likelihood of overall response [odds ratio (OR): 1.64 (95% CI, 1.30–2.18)] and ≥VGPR [2.20 (1.49–3.48)] was associated with a higher cycle 1 belantamab mafodotin average concentration (Supplementary Fig. S3A and S3B). Extramedullary disease was associated with a reduced likelihood of overall response [OR: 0.07 (95% CI, 0.02–0.23)] and ≥VGPR [0.13 (0.03–0.46)], and prior anti-CD38 exposure was associated with a reduced likelihood of ≥VGPR [0.21 (0.08–0.53)]. TTR was not related to cycle 1 belantamab mafodotin exposure.

Exposure–safety analyses showed a greater likelihood of grade ≥3 oARs (per NCI-CTCAE scale) with a higher cycle 1 belantamab mafodotin average concentration [OR: 2.07 (95% CI, 1.45–3.14); Supplementary Fig. S3C]. Similarly, patients who experienced grade ≥2 ocular events had on average higher belantamab mafodotin exposures than those who did not (Supplementary Fig. S4). Higher baseline albumin was associated with a greater likelihood of grade ≥2 oARs [OR: 4.85 (95% CI, 2.20–12.30)]. Time-to-first grade ≥2 or grade ≥3 oARs were not found to be related to cycle 1 belantamab mafodotin average concentration. A greater likelihood and earlier occurrence of grade ≥2 [OR: 2.48 (95% CI, 1.61–4.41)] and ≥3 [OR: 2.08 (95% CI, 1.55–3)] ocular events (per-protocol scale) were found to be associated with a higher cycle 1 belantamab mafodotin average concentration. Dosing schedules were not found to be associated with any of the efficacy or safety endpoints evaluated.

Baseline median sBCMA levels overlapped between responders (range: 15.8–95.9 μg/L) and nonresponders (30.5–181.1 μg/L); nonresponders had higher median baseline sBCMA levels for all but three cohorts (2.5 mg/kg every 6 weeks, 2.5 mg/kg every 3 weeks, and 3.4 mg/kg split every 3 weeks). One patient in the study (in the 3.4 mg/kg split every 3 weeks cohort) developed a treatment-emergent ADA to belantamab mafodotin with no observable impact on safety, efficacy, or PK.

### HRQoL

In patients who responded to the PRO-CTCAE questionnaire, which measured pain symptoms including general pain, headache, muscle pain, and joint pain, the maximum post-baseline PRO-CTCAE scores of 3 or 4 for pain were reported in 54.2% of patients for frequency (frequently/almost constantly; range across cohorts: 33%–88%), 44.9% for severity (severe/very severe; range across cohorts: 18%–75%), and 40.4% for interference (quite a bit/very much; range across cohorts: 27%–56%); no trends in scores were observed across the cohorts.

For HRQoL outcomes related to oAR, baseline median OSDI vision-related function score ranged from 0 to 4.2 across cohorts. For worst-case post-baseline score, 69 (64%) patients experienced a clinically meaningful deterioration (≥12.5-point increase in OSDI score) in vision-related function (range across cohorts: 58%–100%, Supplementary Table S12). The median worst-case post-baseline change in OSDI total scores (range) across cohorts was 16.3 (0–85; 2.5–1.9 mg/kg S/D every 6 weeks) to 51.9 (27–94; 3.4 mg/kg split every 3 weeks). At baseline, the median NEI-VFQ-25 overall composite score ranged from 93.9 to 98.2 across cohorts. The median worst-case post-baseline actual score ranged from 39.9 to 76.5 across cohorts, corresponding to a change in overall composite score from baseline of −49.3 (3.4 mg/kg split) to −9.3 (1.9 mg/kg every 3 weeks; representing a deterioration). The smallest deterioration was seen in the 1.9 mg/kg every 3 weeks cohort [−9.3 (range: −72 to 0)].

## Discussion

DREAMM-6 arm B evaluated the safety, clinical activity, PK, exposure–response, and HRQoL of eight different dosing cohorts of BVd across patients with RRMM who had received ≥1 prior LOT (median = 4). This study enrolled patients throughout the COVID-19 pandemic, and the impact on study and/or treatment discontinuation and study visits was minimal, with only one patient in the 2.5 mg/kg split every 3 weeks cohort discontinuing the study due to the pandemic.

No new safety signals or DLTs were reported, and no clinically meaningful differences in the overall safety and HRQoL profiles were observed between cohorts, indicating the feasibility of this treatment combination. All dosing schedules had an acceptable safety profile. Keratopathy, thrombocytopenia, and reduced platelet count were the most common grade 3 or 4 AEs. Infections were reported (any grade, 42%–89% across cohorts) but were not generally considered SAEs. The risk of serious infections resulting from treatment was reported to be lower with ADCs than for bispecific antibodies for chimeric antigen receptor T cells (24). The majority of patients experienced AEs that were managed with dose reductions or delays. Ocular AEs are a class effect of MMAF-containing ADCs (25), which have been reported across belantamab mafodotin monotherapy and combination studies, and are managed with dose modifications (9, 10, 26, 27). Keratopathy was reported in more than half of all patients, but few patients discontinued treatment because of keratopathy [3 (3%)], and there were no other ocular-related AEs that led to discontinuation; most discontinuations due to AEs were related to neuropathy. Overall, 63% (105/168) of all grade ≥2 ocular events had resolved by the final analysis, demonstrating that these events were generally reversible. Grade ≥2 ocular events were reported in 90% of patients, and these were ongoing in 60 patients (66% of affected patients) at the last follow-up. Overall, the safety findings were generally consistent with the phase III DREAMM-7 study (9).

Belantamab mafodotin dosing and schedules across all eight cohorts demonstrated deep antimyeloma activity for the combination in patients with RRMM: ORRs with the combination (50%–92%) were higher than those reported with belantamab mafodotin monotherapy (32%–41%; refs. 15, 28). Cohorts with a starting dose of 2.5 mg/kg dosing generally achieved the highest proportions of patients with ≥VGPR. The planned dosing regimen of the 2.5 mg/kg every 3 weeks cohort was equivalent to the intended dosing used in the DREAMM-7 study (9), although differences in dose-modification protocols between the studies may have affected exposure to belantamab mafodotin. Rates of ≥VGPRs were similar between the 2.5 mg/kg every 3 weeks cohort (67% at median 16.8 months follow-up) and the BVd arm of DREAMM-7 (66.3% at median 39.4 months follow-up; ref. 12). In the present study, DoR and PFS across cohorts reached up to 29.7 and 24.2 months, respectively. Efficacy data were exploratory and were based on small sample sizes for each cohort with variable durations of follow-up (median DoR was not reached in four cohorts because of relatively short follow-up periods). Efficacy findings were broadly consistent with arm A of DREAMM-6 as well as other belantamab mafodotin combination studies (10, 14, 29). In DREAMM-7, 35.8% achieved ≥CR, with a median DoR of 40.8 months and a median PFS2 not reached (NR; 95% CI, 45.6 months–NR; refs. 11, 12). There was a greater proportion of patients in early lines of RRMM therapy in the DREAMM-7 study compared with DREAMM-6 arm B who were more heavily pretreated (median 4.0 prior LOTs vs. 1.0 in DREAMM-7), and a larger proportion of the study population had received a prior daratumumab (45% in DREAMM-6 arm B vs. 1% in DREAMM-7; ref. 9), which likely contributed to the lower response rates reported in this study. Regardless, the DREAMM-6 arm B study showed that BVd provided clinical benefit in a patient population of which 90% had already received prior bortezomib treatment, although data on prior refractoriness to bortezomib were not collected.

Although this study was not powered to recommend a particular dose, the proportions of patients discontinuing study treatment due to AEs were lower in the 1.9 mg/kg cohorts compared with the 2.5 and 3.4 mg/kg cohorts, indicating a general trend toward more favorable safety profiles in cohorts with lower doses. Deeper responses were observed for the higher starting doses of 2.5 and 3.4 mg/kg cohorts compared with the 1.9 mg/kg cohorts, although the sample size was too small to draw definitive conclusions. The 2.5 mg/kg every 3 weeks schedule balances the efficacy of the 2.5 mg/kg starting dose and safety profile that is manageable and permits dose reductions to 1.9 mg/kg or dose interval extensions as needed for treatment-emergent AEs (30). The efficacy and safety profile of the BVd dosing schedule in DREAMM-7 (belantamab mafodotin 2.5 mg/kg every 3 weeks) and dose-modification protocol (9) broadly align with the findings from this study: Differences in efficacy measures such as PFS between these studies likely reflect the more heavily pretreated population in the DREAMM-6 arm B study. Other belantamab mafodotin combination regimens with different doses and schedules continue to be investigated (31, 32).

This study demonstrated a trend of increased exposure with increased belantamab mafodotin dosing, although peak and average belantamab mafodotin concentrations largely overlapped among cohorts, with patients across five cohorts having dose reductions over time. Exposure–efficacy analyses supported a greater likelihood of response and ≥VGPR with greater cycle 1 belantamab mafodotin exposure. Similarly, exposure–safety analyses showed that greater cycle 1 exposure to belantamab mafodotin was associated with a greater likelihood of grade ≥2 and grade ≥3 ocular events. Despite the associations between cycle 1 exposures and efficacy/safety, dosing schedule had no significant effect on either efficacy or safety.

Similar to the DREAMM-6 arm A findings (14), median baseline sBCMA levels overlapped between responders and nonresponders in each cohort; however, median baseline sBCMA was higher in nonresponders in five cohorts. Separate analyses have shown a decrease in sBCMA levels immediately after belantamab mafodotin infusion regardless of response, with levels remaining measurable and returning to near-baseline in both responders and nonresponders, though levels were quantitatively lower in responders (33). This indicates that the baseline sBCMA levels observed in the current analysis would be expected to remain similar between responders and nonresponders throughout belantamab mafodotin treatment. Further studies are needed to understand the impact of belantamab mafodotin on sBCMA levels and clinical implications for downstream BCMA-targeting therapies.

### Limitations

This study assessed multiple doses and schedules; findings are limited because of the small number of patients in each cohort and the large range in treatment duration across cohorts. Patients were sequentially assigned to cohorts without stratification by baseline characteristics which may have led to bias. Regardless, the findings support the safety and efficacy of BVd and are largely consistent with those reported in the DREAMM-7 phase III trial (9). Additionally, this trial enrolled and treated patients through the COVID-19 pandemic which resulted in protocol deviations in 17 patients, but overall the impact on study discontinuation, treatment discontinuation, and study visits was minimal.

### Conclusion

BVd demonstrated a manageable safety profile with high antimyeloma activity across various belantamab mafodotin dosing cohorts in patients with RRMM who have received a median of 4 LOTs (50% anti-CD38 exposed), a population in need of novel therapeutic options.

## Supplementary Material

Supplementary Methods S1 Supplementary Methods S1

Supplementary Figure S1 Supplementary Figure S1. Study design

Supplementary Figure S2 Supplementary Figure S2. Patient disposition

Supplementary Figure S3 Supplementary Figure S3. Exposure-response relationships.* Probability of an overall response (A), VGPR+ (B), and Grade ≥3 oAEs (NCI-CTCAE) (C) by belantamab mafodotin exposure in Cycle 1 (population PK analysis)

Supplementary Figure S4 Supplementary Figure S4. Probability of Grade ≥2 ophthalmic examination findings (per protocol) by belantamab mafodotin exposure in Cycle 1 (population PK analysis)

Supplementary Table S1 Supplementary Table S1. Full patient eligibility criteria

Supplementary Table S2 Supplementary Table S2. Belantamab mafodotin dose-finding criteria for the modified Toxicity Probability Interval method

Supplementary Table S3 Supplementary Table S3. Covariates for exposure-response analyses

Supplementary Table S4 Supplementary Table S4. Protocol-defined scale for grading corneal events associated with belantamab mafodotin

Supplementary Table S5 Supplementary Table S5. Representativeness of study population

Supplementary Table S6 Supplementary Table S6. Additional patient demographics and clinical characteristics

Supplementary Table S7 Supplementary Table S7. Belantamab mafodotin dose reductions and dose delays

Supplementary Table S8 Supplementary Table S8. Ocular and non-ocular safety summary

Supplementary Table S9 Supplementary Table S9. COVID-19

Supplementary Table S10 Supplementary Table S10 Summary of MRD negativity rate for patients with ≥VGPR

Supplementary Table S11 Supplementary Table S11. Summary of belantamab mafodotin (ADC) non-compartmental PK parameters at Cycle 1 Day 1, Cycle 1 Day 8 (split cohorts only) and during Cycle 1.

Supplementary Table S12 Supplementary Table S12. Summary of patients with ≥12.5-point deterioration from baseline in the OSDI visual related function subscale score

## Data Availability

GSK makes available anonymized individual participant data and associated documents from interventional clinical studies that evaluate medicines, upon approval of proposals submitted to: https://www.gsk-studyregister.com/en.
