# Supplementary material for: Efficacy and Safety of Belantamab Mafodotin with Bortezomib plus Dexamethasone in Patients with Relapsed/Refractory Multiple Myeloma: The DREAMM-6 Arm B Trial
Source: Clin Cancer Res. 2026 Mar 2;32(10):1962–72. doi: 10.1158/1078-0432.CCR-25-3216 (PMC13176820; doi:10.1158/1078-0432.CCR-25-3216)
Supplement: Supplementary Methods S1 [file ccr-25-3216_supplementary_methods_s1_suppms1.pdf]

Supplementary Methods S1

Institutional and/or licensing committee approvals

Australia

Bellberry Human Research Ethics Committees; South Metropolitan Health Service Human Research Ethics Committee (RGO); Peter MacCallum Cancer Centre Ethics Committee (RGO); Central Adelaide Local Health Network Incorporated operating as The Queen Elizabeth Hospital (RGO); University of Wollongong and Southeastern Sydney and Illawarra Area Health Service Health; St Vincent's Hospital, HREC D (RGO); The Alfred Hospital Research Governance (RGO).

Canada

McGill University Health Center-Research Ethics Board.

France

Comité de Protection des Personnes Ile de France II.

Spain

CEIC Hospital Universitario Madrid Monteprincipe.

United Kingdom

Southwest - Central Bristol Research Ethics Committee.

United States

Western Institutional Review Board; Michigan State University Biomedical and Health Institutional Review Board; Greenville Health System Institutional Review Board; Memorial Sloan-Kettering Cancer Center Institutional Review Board; Biomedical Research Alliance of New York, LLC (BRANY); Copernicus Group Institutional Review Board; Washington University in Saint Louis Institutional Review Board; Mary Crowley Medical Research Center Institutional Review Board.

Bortezomib and dexamethasone dosing schedule

Bortezomib 1.3 mg/m^2 was administered SC or IV depending on patients' and institutional preference), and was dosed on Days 1, 4, 8, and 11 of every 21-day cycle for up to 8 cycles. Bortezomib was administered after belantamab mafodotin. Bortezomib doses were calculated based on the participant's body surface area, ranging from a recommended starting dose of 1.3 mg/m^2 to a reduced dose of 1.0 mg/m^2 or 0.7 mg/m^2. Infrequently, if patients experienced an injection-related reaction during belantamab mafodotin administration, the bortezomib

administration was delayed until the reaction had resolved and the patient was considered clinically stable.

Dexamethasone was administered at 20 mg orally (PO) or IV on Days 1, 2, 4, 5, 8, 9, 11, and 12 of every 21-day cycle for up to 8 cycles. Dexamethasone was administered at a fixed dose of 20 mg PO or IV, with no adjustments needed for body weight or body surface area. Dexamethasone dose could be reduced at the investigator's discretion for patients aged >75 years, for those with a body mass index <18.5 kg/m^2, or for patients who had previous unacceptable side effects associated with glucocorticoid therapy.

The dosing bortezomib and dexamethasone schedules were the same across all cohorts.

Belantamab mafodotin modified Toxicity Probability Interval (mTPI) design

In the dose-escalation phase, three belantamab mafodotin dose levels (1.9, 2.5, and 3.4 mg/kg) were evaluated in combination with fixed doses of bortezomib and dexamethasone. Cohorts were recruited in blocks of 3 patients. Patients were entered in a staggered approach with ≥1 day between each patient's first dose of belantamab mafodotin, to minimize the risk of inadvertently exceeding the maximum tolerated dose (MTD) in multiple patients. The safety data over the first cycle for each patient at a given dose level was evaluated for ≥3 patients before it was decided to enroll additional patients at the subsequent dose level.

Patients who received ≥1 full dose of belantamab mafodotin and ≥75% of planned doses of bortezomib and dexamethasone by the end of the first cycle were evaluated for DLTs.
The mTPI rules are detailed in **Supplementary Table S2**. Using these rules, if the decision at the belantamab mafodotin 2.5 mg/kg dose was to stay at the same dose but <6 patients had been evaluated for DLT, an additional 3 patients were enrolled for DLT evaluation; if it was decided to stay at the same dose after these additional patients, the dose escalation was complete. If the decision at the belantamab mafodotin 2.5 mg/kg dose was to de-escalate the dose or if there was unacceptable toxicity, the lower 1.9 mg/kg could be evaluated. If the 1.9 mg/kg dose was not tolerated, the trial would be terminated early. If the decision at the belantamab mafodotin 3.4 mg/kg dose was to escalate dose or stay at the same dose and <6 patients had been evaluated for DLTs at that dose (and the planned maximum sample size was not reached), an additional 3 patients were evaluated for DLTs at that dose. If it was decided to stay at the same dose or escalate after these additional patients, the dose escalation was

complete. Otherwise, dose escalations continued until the maximum sample size (12 DLT-evaluable patients for each treatment) was reached.

DLT criteria

DLT were assessed using NCI-CTCAE v4.03 and included at least one of the following: Grade ≥3 febrile neutropenia lasting >48 hours despite adequate treatment; Grade 4 thrombocytopenia <25,000/mm^3 accompanied by clinically significant bleeding; any Grade ≥3 non-hematologic toxicity (other than ocular events) that was more severe than expected with belantamab mafodotin treatment or did not resolve with appropriate supportive treatment within 48 hours; any Grade ≥3 non-hematologic laboratory value if the laboratory abnormality persisted for >48 hours despite supportive treatment or the abnormality led to hospitalization; any Grade 4 ocular event; or liver toxicity meeting a predefined stopping criteria.

Protocol amendments

Three amendments to the study protocol were made:

1. Key changes made in the first amendment included a minor correction to a patient eligibility criterion (exclusion of patients with prior allogeneic stem cell transplant to avoid unpredictable toxicity) and clarification of scenarios for study drug dose delays after Cycle 1 to provide guidance on dose interruptions and reductions.
2. For the second protocol amendment, following a review of the safety data from DREAMM-2, which indicated ocular changes associated with belantamab mafodotin treatment were mostly limited to the corneal epithelium (1,2), subsequent collection of ocular safety data was focused on corneal changes, and the use of corticosteroid eye drops as prophylactic treatment was discontinued due to evidence that this treatment is ineffective in preventing or mitigating changes to the corneal epithelium (3). In addition, to reduce increased exposure of belantamab mafodotin over time and to potentially improve the benefit/risk for patients, two reduced-dose cohorts – namely 1.9 mg/kg Q3W and 1.9 mg/kg Q6W – were added. Also, extended dosing schedules for belantamab mafodotin were introduced (1.9 mg/kg Q6W and 2.5–1.9 mg/kg S/D Q6W cohorts).
3. The third amendment to the protocol was made to update the definition of the end of study and to include continued details on study intervention access after the final analysis.

PRO-CTCAE

The PRO-CTCAE evaluates symptomatic treatment side effects and toxicity. AE scores were scaled from 0 to 4 depending on frequency (never, rarely, occasionally, frequently, almost constantly), severity (none, mild, moderate, severe, very severe) and interference (not at all, a little bit, somewhat, quite a bit, very much).

OSDI

The OSDI is a 12-item questionnaire designed to assess the frequency of dry eye symptoms and their impact on vision-related functioning, including reading and driving (4); a ≥12.5-point decrease in score indicates an improvement in vision-related functioning; conversely, a ≥12.5-point increase in score indicates a deterioration in vision-related functioning.

NEI-VFQ-25

The NEI-VFQ-25 consists of a base set of 25 vision-targeted questions representing 11 vision-related constructs (global vision rating; difficulty with near or distant vision activities; limitations in social functioning due to vision, limitations in role functioning due to vision; dependency on others due to vision; mental health symptoms due to vision; driving difficulties; limitations with peripheral vision; limitations with color vision; ocular pain) plus an additional single-item general health rating question (5). An increase in scores (positive change) from baseline indicates an improvement in vision-related constructs.

PK statistical considerations

Linear and semi-logarithmic individual concentration–time profiles and mean and median profiles were plotted for belantamab mafodotin. Concentrations of belantamab mafodotin were summarized by planned timepoint (and dose level for belantamab mafodotin). Concentration of bortezomib was determined in plasma samples using validated bioanalytical methods and summarized by planned timepoint.

The population PK analysis was performed using NONMEM software, version 7.3.0 (ICON Development Solutions), and run management was performed using Pirana (version 2.9.7). All post-processing of population PK results and exposure-response analyses were performed using R (version 3.2.5 or higher). Population and individual participant-predicted ADC concentrations were generated using MAXEVALS option set to zero in NONMEM.

Exposure-response analyses

All post-processing of exposure-response analyses were performed using R (version 3.2.5 or higher).

Exposure-response analyses were performed utilizing Cycle 1 exposure estimates for belantamab mafodotin (C_{avg}), using the individual post hoc Cycle 1 PK parameter estimates obtained from the population PK analysis (6).

Response refers to endpoints related to efficacy or AESIs. Exposure-response analyses for efficacy were conducted using belantamab mafodotin ADC exposure and performed for the probability of response. Exposure-response analyses for safety were undertaken using belantamab mafodotin ADC exposure and performed for the probability of any and time to first Grade ≥2 or ≥3 oAE, assessed per NCI-CTCAE, and ophthalmic examination findings.

Patients were included in exposure-safety analyses if they received at least one dose of belantamab mafodotin and had a measurable PK sample available. Patients were included in the exposure-efficacy analyses population if, in addition to the above, they had measurable disease at baseline. Exposure-response analyses were not performed for endpoints with low (≤10%) or high (≥90%) incidence rate.

Logistic regression models were used to determine the relationship between ADC exposure measures and the probability of response. Covariate analyses (listed in **Supplementary Table S3**) were performed for logistic regression models using the stepwise forward addition and backward elimination procedure. Covariates meeting α of 0.01 in the univariate step were included in the stepwise covariate search. After the full model was defined, the significance of each covariate was tested individually by removal one at a time from the full model. The elimination steps were repeated until all non-significant covariates were excluded, and the final model was defined.

References

1. Farooq AV, Degli Esposti S, Popat R, Thulasi P, Lonial S, Nooka AK*, et al.* Corneal Epithelial Findings in Patients with Multiple Myeloma Treated with Antibody-Drug Conjugate Belantamab Mafodotin in the Pivotal, Randomized, DREAMM-2 Study. *Ophthalmol Ther* **2020**;9:889-911.

2. Nooka AK, Cohen AD, Lee HC, Badros A, Suvannasankha A, Callander N*, et al.* Single-agent belantamab mafodotin in patients with relapsed/refractory multiple myeloma: Final analysis of the DREAMM-2 trial. *Cancer* **2023**;129:3746-60.
3. Lonial S, Lee HC, Badros A, Trudel S, Nooka AK, Chari A*, et al.* Belantamab mafodotin for relapsed or refractory multiple myeloma (DREAMM-2): a two-arm, randomised, open-label, phase 2 study. *Lancet Oncol* **2020**;21:207-21.
4. Dougherty BE, Nichols JJ, Nichols KK. Rasch analysis of the Ocular Surface Disease Index (OSDI). *Invest Ophthalmol Vis Sci* **2011**;52:8630-5.
5. Mangione CM, Lee PP, Gutierrez PR, Spritzer K, Berry S, Hays RD. Development of the 25-item National Eye Institute Visual Function Questionnaire. *Arch Ophthalmol* **2001**;119:1050-8.
6. Papathanasiou T, Kaullen J, Polireddy K, Chen X, Ho YL, Taylor A*, et al.* Population Pharmacokinetics for Belantamab Mafodotin Monotherapy and Combination Therapies in Patients with Relapsed/Refractory Multiple Myeloma. *Clin Pharmacokinet* **2025**;64:925-42.
