# Supplementary material for: Efficacy and Safety of Belantamab Mafodotin with Bortezomib plus Dexamethasone in Patients with Relapsed/Refractory Multiple Myeloma: The DREAMM-6 Arm B Trial
Source: Clin Cancer Res. 2026 Mar 2;32(10):1962–72. doi: 10.1158/1078-0432.CCR-25-3216 (PMC13176820; doi:10.1158/1078-0432.CCR-25-3216)
Supplement: Supplementary Figure S1 — Study design [file ccr-25-3216_supplementary_figure_s1_suppfs1.pdf]

Supplementary Figure S1. Study design

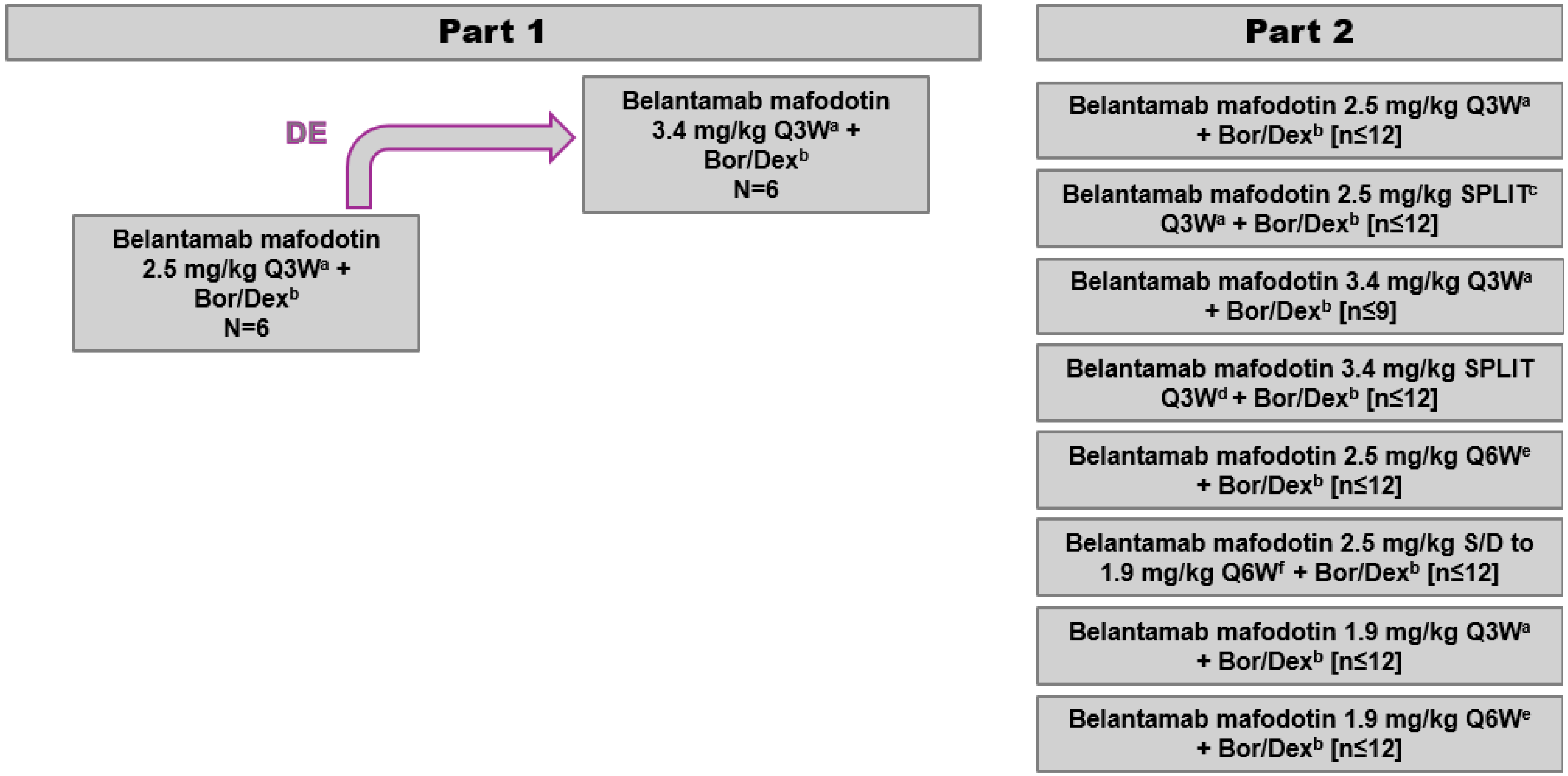

Combination therapy continued for up to 8 combination cycles; belantamab mafodotin further continued until progressive disease, death, intolerable toxicity, or consent withdrawn. Cohorts followed-up for progression-free survival and overall survival.
Number of patients in each cohort varied from 12 to 18.

[a]Full assigned dose of belantamab mafodotin (1.9 mg/kg, 2.5 mg/kg, or 3.4 mg/kg) administered on Day 1 of any 21-day cycle; [b]bortezomib
(1.3 mg/m^2 on Days 1, 4, 8, and 11) + dexamethasone (20 mg on Days 1, 2, 4, 5, 8, 9, 11, and 12) of any 21-day cycle; [c]split belantamab mafodotin dose of 1.25 mg/kg belantamab mafodotin administered on Day 1 and Day 8 of any 21-day cycle; [d]split belantamab mafodotin dose of 1.7 mg/kg belantamab mafodotin dose administered on Day 1 and Day 8 of any 21-day cycle; [e]full belantamab mafodotin dose (1.9 mg/kg or 2.5 mg/kg) administered on Day 1 of any 42-day cycle; [f]2.5 mg/kg belantamab mafodotin dose administered at C1D1 followed by a S/D to
1.9 mg/kg belantamab mafodotin dose administered on Day 1 of 42-day cycles C2.

Bor, bortezomib; C, cycle; DE, dose escalation; Dex, dexamethasone; Q3W, every 3 weeks; Q6W, every 6 weeks; S/D, step-down.
