# Supplementary material for: Efficacy and Safety of Belantamab Mafodotin with Bortezomib plus Dexamethasone in Patients with Relapsed/Refractory Multiple Myeloma: The DREAMM-6 Arm B Trial
Source: Clin Cancer Res. 2026 Mar 2;32(10):1962–72. doi: 10.1158/1078-0432.CCR-25-3216 (PMC13176820; doi:10.1158/1078-0432.CCR-25-3216)
Supplement: Supplementary Figure S2 — Patient disposition [file ccr-25-3216_supplementary_figure_s2_suppfs2.pdf]

Supplementary Figure S2. Patient disposition

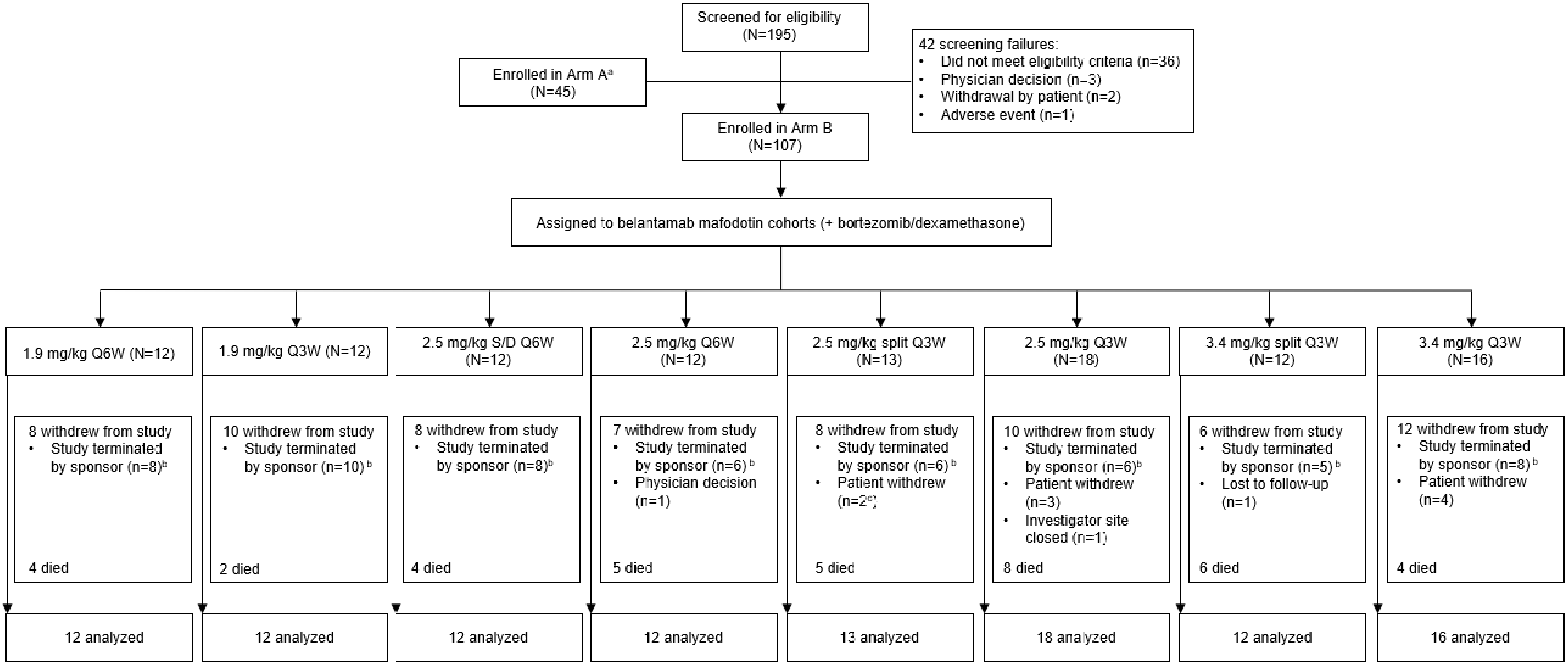

[a]Reported separately (1). One Patient who was eligible for enrolment was not treated; [b]study terminated is indicative of patients continuing into post analysis continued treatment, which includes patients who were ongoing as well as those that were in survival follow-up at the time of last patient last visit. The primary reason for withdrawal was continuation into post analysis continued treatment for all but 11 patients who withdrew for the study (1 physician decision, 9 patient decision, 1 lost to follow-up); [c]1 patient withdrawal was related to COVID-19.

Q3W, every 3 weeks; Q6W, every 6 weeks; S/D, step-down.

References

1. Popat R, Augustson B, Gironella M, Lee C, Cannell P, Patel N*, et al.* Results from Arm A of Phase 1/2 DREAMM-6 trial: belantamab mafodotin with lenalidomide plus dexamethasone in patients with relapsed/refractory multiple myeloma. *Blood Cancer J* **2024**;14:184.
