# Supplementary material for: Efficacy and Safety of Belantamab Mafodotin with Bortezomib plus Dexamethasone in Patients with Relapsed/Refractory Multiple Myeloma: The DREAMM-6 Arm B Trial
Source: Clin Cancer Res. 2026 Mar 2;32(10):1962–72. doi: 10.1158/1078-0432.CCR-25-3216 (PMC13176820; doi:10.1158/1078-0432.CCR-25-3216)
Supplement: Supplementary Figure S3 — Exposure-response relationships.* Probability of an overall response (A), VGPR+ (B), and Grade ≥3 oAEs (NCI-CTCAE) (C) by belantamab mafodotin exposure in Cycle 1 (population PK analysis) [file ccr-25-3216_supplementary_figure_s3_suppfs3.pdf]

Supplementary Figure S3. Exposure-response relationships.* Probability of an overall response (A), VGPR+ (B), and Grade ≥3 oAEs (NCI-CTCAE) (C) by belantamab mafodotin exposure in Cycle 1 (population PK analysis)

A

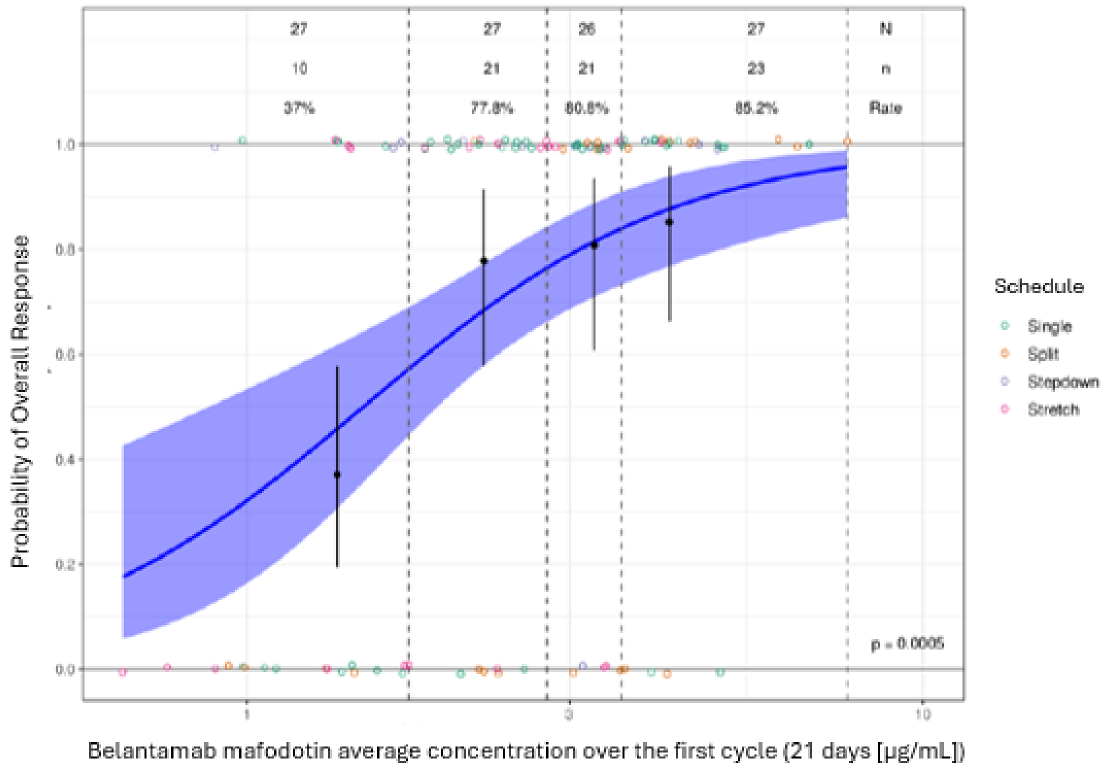

Q1: 1.36 (0.655, 1.74), Q2: 2.24 (1.83, 2.78), Q3: 3.26 (2.79, 3.58), Q4: 4.22 (3.62, 7.74)

B

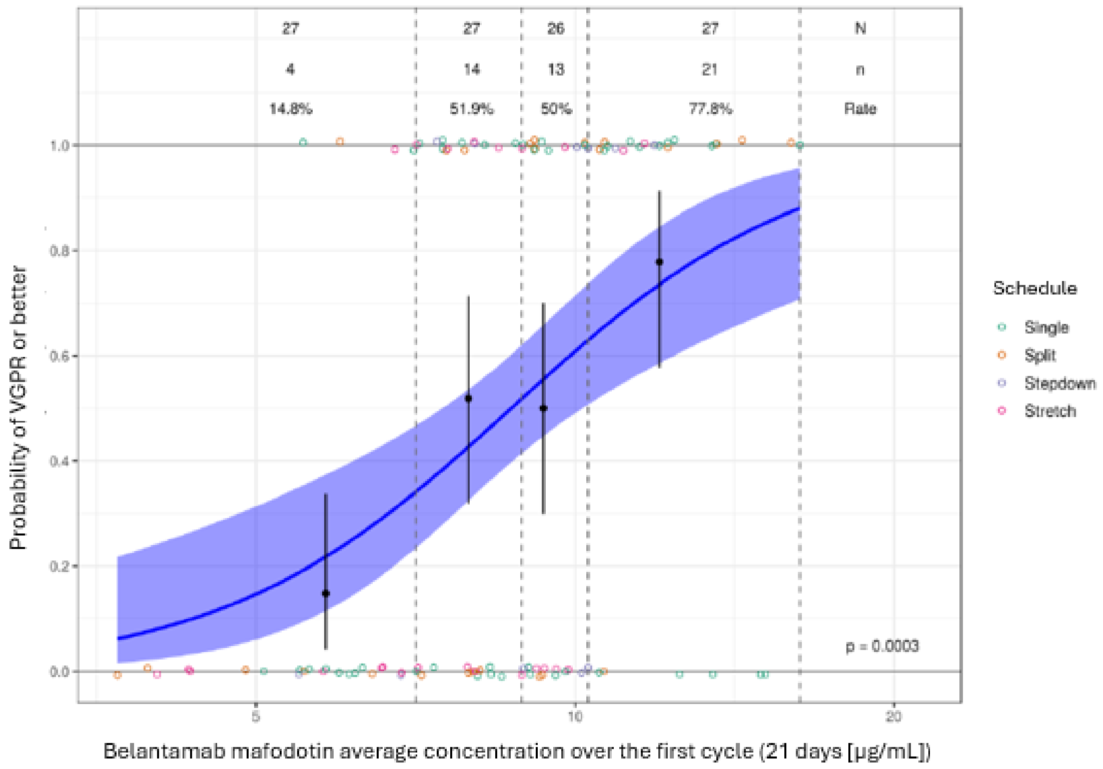

Q1: 5.82 (3.7, 7.08), Q2: 7.94 (7.08, 8.91), Q3: 9.34 (8.91, 10.3), Q4: 12 (10.5, 16.3)

C

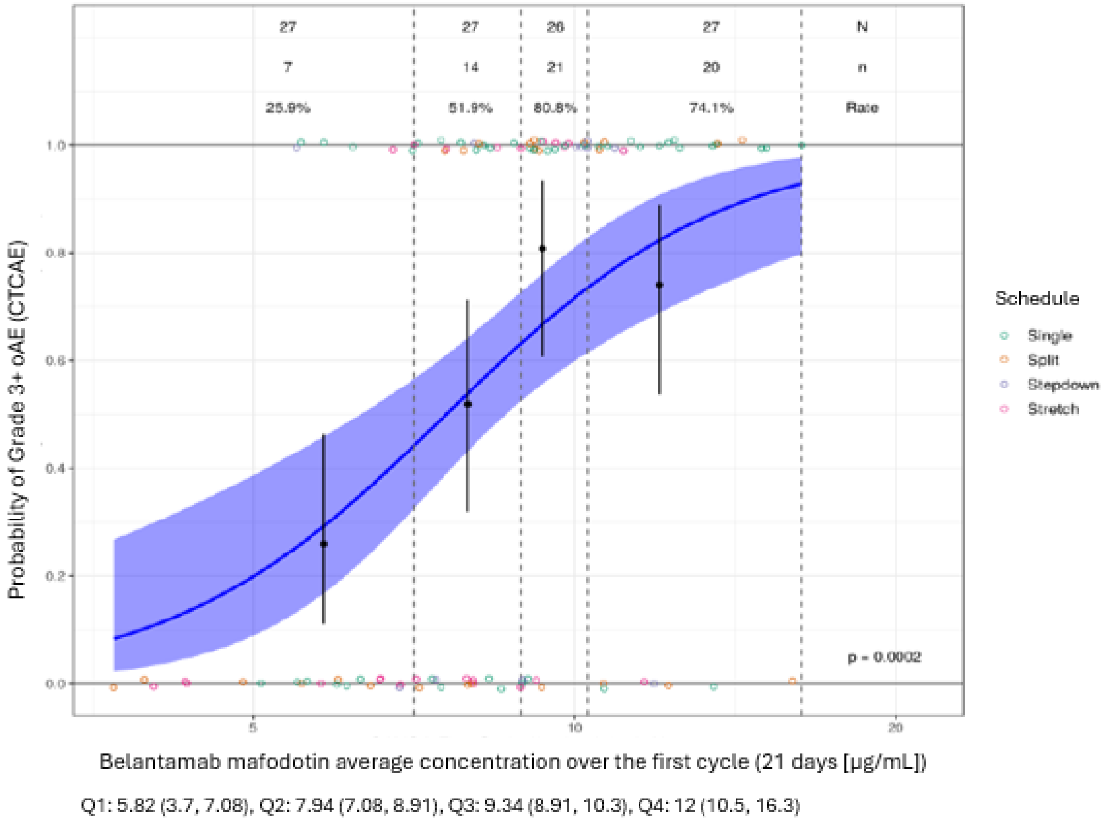

The independent variable was divided into quartiles. Median (min, max) for each quartile are reported in the footnotes. Black points and error bars represent the observed proportions and 95% CI for each quartile (plotted at the median exposure within each quartile), respectively. The blue curve represents the prediction of the univariate logistic regression model; the blue shaded region represents the 95% CI of the prediction.

NCI-CTCAE, National Cancer Institute-Common Toxicity Criteria for Adverse Events; PK, pharmacokinetic; VGPR, very good partial response.
