# Supplementary material for: Efficacy and Safety of Belantamab Mafodotin with Bortezomib plus Dexamethasone in Patients with Relapsed/Refractory Multiple Myeloma: The DREAMM-6 Arm B Trial
Source: Clin Cancer Res. 2026 Mar 2;32(10):1962–72. doi: 10.1158/1078-0432.CCR-25-3216 (PMC13176820; doi:10.1158/1078-0432.CCR-25-3216)
Supplement: Supplementary Figure S4 — Probability of Grade ≥2 ophthalmic examination findings (per protocol) by belantamab mafodotin exposure in Cycle 1 (population PK analysis) [file ccr-25-3216_supplementary_figure_s4_suppfs4.pdf]

Supplementary Figure S4. Probability of Grade ≥2 ophthalmic examination findings (per protocol) by belantamab mafodotin exposure in Cycle 1 (population PK analysis)

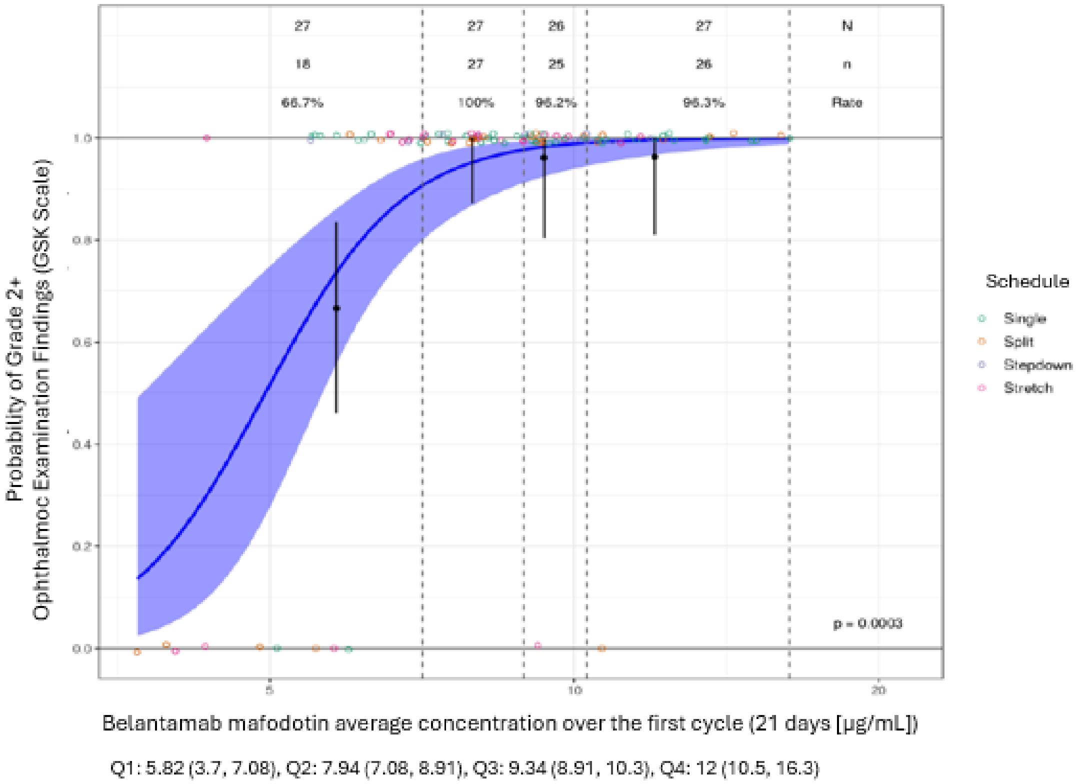

PK, pharmacokinetic.
