# Supplementary material for: Efficacy and Safety of Belantamab Mafodotin with Bortezomib plus Dexamethasone in Patients with Relapsed/Refractory Multiple Myeloma: The DREAMM-6 Arm B Trial
Source: Clin Cancer Res. 2026 Mar 2;32(10):1962–72. doi: 10.1158/1078-0432.CCR-25-3216 (PMC13176820; doi:10.1158/1078-0432.CCR-25-3216)
Supplement: Supplementary Table S1 — Full patient eligibility criteria [file ccr-25-3216_supplementary_table_s1_suppts1.pdf]

Supplementary Table S1. Full patient eligibility criteria

**Inclusion criteria; at screening, patients:**
Were capable of giving signed informed consent and complied with the requirements and restrictions listed in the informed consent form
Were 18 years or older at the time consent was obtained
Had a confirmed MM diagnosis as defined by the International Myeloma Working Group criteria (7)
Had an Eastern Cooperative Oncology Group performance status between 0 and 2
Had prior autologous SCT >100 days before study enrollment, with no active bacterial, viral, or fungal infection(s), or were considered transplant ineligible
Were treated with ≥1 prior LOT and had documented disease progression during or after their most recent therapy
Had measurable MM disease defined by ≥1 of the following criteria:<br>• Urine M-protein excretion ≥200 mg/24 h<br>• Serum M-protein concentration ≥0.5 g/dL<br>• Serum FLC assay FLC level ≥10 mg/dL and an abnormal serum FLC ratio (<0.26 or >1.65)
Prior treatment-related toxicities as defined by NCI-CTCAE, version 4.03, 2010 (8) must have been Grade ≤1 at the time of enrollment, except for alopecia
Had adequate organ system functions as defined by absolute neutrophil count* ≥1.5 × 10^9/L, hemoglobin ≥8.0 g/dL, platelets ≥75 × 10^9/L, total bilirubin ≤1.5 × ULN,† ALT ≤2.5 × ULN, eGFR‡ ≥40 mL/min/1.73 m^2, albumin/creatinine ratio from spot urine ≤500 mg/g, left ventricular ejection fraction by echocardiogram ≥40%
Were either not a woman of childbearing potential or were using a highly effective (failure rate <1%/year) contraceptive method
**Patients not meeting the inclusion criteria were excluded, along with those meeting the following exclusion criteria:**
Had systemic anti-myeloma therapy (including systemic steroids) within ≤14 days, or plasmapheresis within 7 days prior to first dose of study drug
Use of an investigational drug within 14 days or five half-lives (whichever was longer) preceding first dose of study drug
Had prior treatment with a mAb antibody within 30 days of receiving the first belantamab mafodotin dose
Had prior allogenic SCT; patients with a prior syngeneic transplant were allowed provided there was no history of or active graft versus host disease
Had evidence of active mucosal or internal bleeding
Had any major surgery ≤4 weeks before screening
Had presence of active renal condition, except for those with isolated proteinuria due to MM
Had any serious and/or unstable pre-existing medical, psychiatric disorder or other conditions that could interfere with participation in the study
Had a current active liver or biliary disease (except for Gilbert's syndrome or asymptomatic gallstones, or otherwise stable chronic liver disease per investigator's assessment)
Had invasive malignancies, other than MM and curatively treated non-melanoma skin cancer, that required active therapy or were stable for <2 years
Had evidence of cardiovascular risk including ≥1 of the following:<br>• Current clinically significant untreated arrhythmias, including clinically significant electrocardiogram abnormalities such as 2nd degree (Mobitz Type II) or 3rd degree atrioventricular block

- History of myocardial infarction, acute coronary syndromes (including unstable angina), coronary angioplasty, or stenting or bypass grafting ≤3 months before screening
- Class III or IV heart failure as defined by the New York Heart Association functional classification system
- Uncontrolled hypertension

Had a known immediate or delayed hypersensitivity reaction or idiosyncratic reaction to drugs chemically related to belantamab mafodotin, or any of the components of the study treatment

Pregnant or lactating female

Had an active infection requiring treatment, or known HIV infection

Had presence of hepatitis B surface antigen, or hepatitis B core antibody at screening or within 3 months before the first belantamab mafodotin dose

Had a positive hepatitis C antibody test result or positive hepatitis C RNA test result at screening or ≤3 months before the first belantamab mafodotin dose, except those with positive test results due to prior resolved disease

Had current corneal disease except mild punctate keratopathy

Had unacceptable adverse effects from previous bortezomib treatment

Had ongoing Grade 2 or higher peripheral neuropathy or neuropathic pain from previous bortezomib treatment

Intolerance or contraindications to anti-viral prophylaxis

---

*Without growth factor support for the past 14 days, excluding erythropoietin; †isolated bilirubin 1.5 × ULN was acceptable if bilirubin was fractionated, and direct bilirubin was <35%; ‡calculated using the Modified Diet in Renal Disease formula.

eGFR, estimated glomerular filtration rate; FLC, free light chain; HIV, human immunodeficiency virus; LOT, line of therapy; mAb. Monoclonal antibody; MM, multiple myeloma; NCI-CTCAE, National Cancer Institute-Common Toxicity Criteria for Adverse Events; RNA, ribonucleic acid; SCT, stem cell transplant; ULN, upper limit of normal.
