# Supplementary material for: Efficacy and Safety of Belantamab Mafodotin with Bortezomib plus Dexamethasone in Patients with Relapsed/Refractory Multiple Myeloma: The DREAMM-6 Arm B Trial
Source: Clin Cancer Res. 2026 Mar 2;32(10):1962–72. doi: 10.1158/1078-0432.CCR-25-3216 (PMC13176820; doi:10.1158/1078-0432.CCR-25-3216)
Supplement: Supplementary Table S2 — Belantamab mafodotin dose-finding criteria for the modified Toxicity Probability Interval method [file ccr-25-3216_supplementary_table_s2_suppts2.pdf]

Supplementary Table S2. Belantamab mafodotin dose-finding criteria for the modified Toxicity Probability Interval method

		Number of patients treated at current dose					
		1	2	3	4	5	6
Number of patients with DLT	0	E	E	E	E	E	E
	1	D	S	S	S	S	E
	2		DU	D	S	S	S
	3			DU	DU	D	S
	4				DU	DU	DU
	5					DU	DU
	6						DU

Dose-finding decision responses: E, escalate the dose; S, stay at the same dose; D, de-escalate the dose; DU, the current dose level is unacceptable because of high toxicity and should be excluded from the trial, along with any higher dose levels.

DLT, dose-limiting toxicity.

Example of table interpretation: when 1 of 3 participants experiences toxicity, the decision can be located at row 1 and column 3, which is S – to stay at the current dose level. Consequently, the next cohort of participants will be treated at the same dose level currently being used. If zero of three participants experience toxicity, the decision is at row 0 and column 3, which is E – to escalate. Thus, the next cohort of participants will be treated at the next higher dose level. If 3 of 3 participants experience toxicity, the decision is DU – to deescalate to the next-lower dose level and exclude the current dose and any higher dose from the trial, because toxicity levels are unacceptable.
