# Supplementary material for: Efficacy and Safety of Belantamab Mafodotin with Bortezomib plus Dexamethasone in Patients with Relapsed/Refractory Multiple Myeloma: The DREAMM-6 Arm B Trial
Source: Clin Cancer Res. 2026 Mar 2;32(10):1962–72. doi: 10.1158/1078-0432.CCR-25-3216 (PMC13176820; doi:10.1158/1078-0432.CCR-25-3216)
Supplement: Supplementary Table S3 — Covariates for exposure-response analyses [file ccr-25-3216_supplementary_table_s3_suppts3.pdf]

Supplementary Table S3. Covariates for exposure-response analyses

Category	Covariates
Demographics	Weight, body surface area, body mass index, age, race, gender
Baseline clinical status	eGFR in mL/min, albumin, alanine transaminase, aspartate aminotransferase, total bilirubin, C-reactive protein, serum IgG, renal function category, hepatic function category
Baseline disease status	Type of MM (secretory or non-secretory), type of myeloma light chain, type of myeloma Ig, cytogenetics risk, soluble BCMA, β2-microglobulin, lactate dehydrogenase, stage of disease, ECOG status, presence of extramedullary disease
Others	Number of prior lines of therapy, previous anti-CD38 treatment, dosing schedule
Specific to corneal events	History of intraocular surgery, lens status, known history of dry eye per screening questionnaire, presence of keratopathy at baseline examination
Specific to thrombocytopenia	Baseline platelet count

BCMA, B cell maturation antigen; eGFR, estimated glomerular filtration rate; ECOG, Eastern Cooperative Oncology Group; Ig, immunoglobulin; MM, multiple myeloma.
