# Supplementary material for: Efficacy and Safety of Belantamab Mafodotin with Bortezomib plus Dexamethasone in Patients with Relapsed/Refractory Multiple Myeloma: The DREAMM-6 Arm B Trial
Source: Clin Cancer Res. 2026 Mar 2;32(10):1962–72. doi: 10.1158/1078-0432.CCR-25-3216 (PMC13176820; doi:10.1158/1078-0432.CCR-25-3216)
Supplement: Supplementary Table S4 — Protocol-defined scale for grading corneal events associated with belantamab mafodotin [file ccr-25-3216_supplementary_table_s4_suppts4.pdf]

Supplementary Table S4. Protocol-defined scale for grading corneal events associated with belantamab mafodotin

Measure	Grade 1	Grade 2	Grade 3	Grade 4
Ophthalmic examination finding	Mild superficial keratopathy (change from baseline)	Moderate punctate keratopathy *and/or* mild/patchy microcysts *and/or* mild/patchy epithelial or stromal edema *and/or* sub-epithelial haze (peripheral) *and/or* active stromal opacity (peripheral)	Severe punctate keratopathy *and/or* diffuse microcysts *and/or* diffuse epithelial or stromal edema *and/or* sub-epithelial haze (central) *and/or* active stromal opacity (central)	Corneal ulcer
Visual Acuity*	Change of 1 line from baseline	Change of 2–3 lines from baseline and not worse than 20/200	Change of more than 3 lines from baseline and not worse than 20/200	Worse than Vision 20/200

Grading was based on most severe finding; if eyes differed in severity, the protocol-defined grading was based on the more severe eye. *Change in visual acuity due to ocular events, presented as Snellen equivalent and logarithm of the minimum angle of resolution value. If a change in vision was for a reason other than ocular events, the ophthalmic examination findings drove the event grading. If a patient had a baseline visual acuity of 20/200 or worse in an eye, ophthalmic examination findings drove the event grading.
