# Supplementary material for: Efficacy and Safety of Belantamab Mafodotin with Bortezomib plus Dexamethasone in Patients with Relapsed/Refractory Multiple Myeloma: The DREAMM-6 Arm B Trial
Source: Clin Cancer Res. 2026 Mar 2;32(10):1962–72. doi: 10.1158/1078-0432.CCR-25-3216 (PMC13176820; doi:10.1158/1078-0432.CCR-25-3216)
Supplement: Supplementary Table S5 — Representativeness of study population [file ccr-25-3216_supplementary_table_s5_suppts5.pdf]

Supplementary Table S5. Representativeness of study population

Cancer Type/subtype/stage/condition	Multiple myeloma/relapsed and refractory
Sex	Men have a higher incidence of multiple myeloma that women (1.5:1) (1).
Age	Median age at diagnosis is between 66 and 70 years, with incidence increasing with age (1,2).
Race/ethnicity	Black people have a higher incidence of MM than White people (2:1) (1-3).
Geography	Australasia, Northern America and western Europe have a higher incidence of MM than the rest of the world (4).
Overall representativeness of this study	In DREAMM-6 Arm B, which collected patients' demographics reported by the investigator at enrollment, the proportion of male patients and median age (66.0 years) were within the range of the multiple myeloma population. African American patients (8%) were underrepresented in this trial where White patients were predominant (82%). Geographically, this study recruited patients from Australia, Canada, Spain, United Kingdom, and the US, including countries with the highest age standardized incidence of multiple myeloma worldwide.

References

1. Padala SA, Barsouk A, Barsouk A, Rawla P, Vakiti A, Kolhe R*, et al.* Epidemiology, Staging, and Management of Multiple Myeloma. *Med Sci (Basel)* **2021**;9.
2. Kazandjian D. Multiple myeloma epidemiology and survival: A unique malignancy. *Semin Oncol* **2016**;43:676-81.
3. Waxman AJ, Mink PJ, Devesa SS, Anderson WF, Weiss BM, Kristinsson SY*, et al.* Racial disparities in incidence and outcome in multiple myeloma: a population-based study. *Blood* **2010**;116:5501-6.

4. Mafra A, Laversanne M, Marcos-Gragera R, Chaves HVS, McShane C, Bray F*, et al.* The global multiple myeloma incidence and mortality burden in 2022 and predictions for 2045. *J Natl Cancer Inst* **2024**.
