# Supplementary material for: Efficacy and Safety of Belantamab Mafodotin with Bortezomib plus Dexamethasone in Patients with Relapsed/Refractory Multiple Myeloma: The DREAMM-6 Arm B Trial
Source: Clin Cancer Res. 2026 Mar 2;32(10):1962–72. doi: 10.1158/1078-0432.CCR-25-3216 (PMC13176820; doi:10.1158/1078-0432.CCR-25-3216)
Supplement: Supplementary Table S6 — Additional patient demographics and clinical characteristics [file ccr-25-3216_supplementary_table_s6_suppts6.pdf]

Supplementary Table S6. Additional patient demographics and clinical characteristics

Parameter	1.9 mg/kg Q6W<br><br>n=12	1.9 mg/kg Q3W<br><br>n=12	2.5–1.9 mg/kg S/D Q6W<br><br>n=12	2.5 mg/kg Q6W<br><br>n=12	2.5 mg/kg split Q3W<br><br>n=13	2.5 mg/kg Q3W<br><br>n=18	3.4 mg/kg split Q3W<br><br>n=12	3.4 mg/kg Q3W<br><br>n=16	All treated<br><br>N=107
**Prior MM treatment, n (%)**									
Corticosteroids	12 (100)	12 (100)	12 (100)	12 (100)	13 (100)	18 (100)	12 (100)	16 (100)	107 (100)
Chemotherapy	12 (100)	12 (100)	12 (100)	10 (83)	13 (100)	15 (83)	12 (100)	16 (100)	102 (95)
Proteasome inhibitor	12 (100)	12 (100)	11 (92)	10 (83)	13 (100)	17 (94)	11 (92)	16 (100)	102 (95)
Bortezomib	11 (92)	11 (92)	11 (92)	8 (67)	13 (100)	16 (89)	11 (92)	15 (94)	96 (90)
Immunomodulator	12 (100)	10 (83)	11 (92)	12 (100)	12 (92)	17 (94)	11 (92)	15 (94)	100 (93)
Lenalidomide	11 (92)	9 (75)	9 (75)	11 (92)	11 (85)	15 (83)	8 (67)	12 (75)	86 (80)
mAb	5 (42)	7 (58)	7 (58)	6 (50)	7 (54)	11 (61)	9 (75)	5 (31)	57 (53)
Daratumumab	5 (42)	6 (50)	6 (50)	5 (42)	6 (46)	9 (50)	7 (58)	4 (25)	48 (45)

Isatuximab	0 (0)	1 (8)	1 (8)	1 (8)	1 (8)	0 (0)	0 (0)	1 (6)	5 (5)
Other	0 (0)	0 (0)	2 (17)	2 (17)	1 (8)	1 (6)	4 (33)	3 (19)	13 (12)
**ECOG performance score, n (%)**									
0	4 (33)	9 (75)	7 (58)	4 (33)	7 (54)	8 (44)	6 (50)	8 (50)	53 (50)
1	8 (67)	2 (17)	5 (42)	8 (67)	3 (23)	7 (39)	5 (42)	8 (50)	46 (43)
2	0 (0)	1 (8)	0 (0)	0 (0)	3 (23)	3 (17)	1 (8)	0 (0)	8 (7)
**Extramedullary disease present, n (%)[b]**	2 (17)	2 (17)	2 (17)	1 (8)	3 (23)	5 (28)	3 (25)	4 (25)	22 (21)
**Lytic bone lesions present, n (%)**	7 (58)	6 (50)	10 (83)	10 (83)	6 (46)	15 (83)	7 (58)	10 (63)	71 (66)
**Myeloma Ig, n (%)**									
IgA	5 (42)	5 (42)	1 (8)	1 (8)	5 (38)	5 (28)	1 (8)	0 (0)	23 (21)
IgD	1 (8)	0 (0)	1 (8)	0 (0)	0 (0)	0 (0)	0 (0)	0 (0)	2 (2)
IgG	8 (67)	6 (50)	9 (75)	8 (67)	7 (54)	10 (56)	7 (58)	16 (100)	71 (66)
IgM	0 (0)	0 (0)	0 (0)	0 (0)	0 (0)	1 (6)	1 (8)	0 (0)	2 (2)

Not applicable	0 (0)	1 (8)	1 (8)	3 (25)	2 (15)	4 (22)	3 (25)	0 (0)	14 (13)
**Myeloma light chain, n (%)**									
No	4 (33)	5 (42)	7 (58)	4 (33)	2 (15)	3 (17)	0 (0)	3 (19)	28 (26)
Yes: Kappa light chain	4 (33)	4 (33)	4 (33)	4 (33)	7 (54)	11 (61)	7 (58)	6 (38)	47 (44)
Yes: Lambda light chain	4 (33)	3 (25)	1 (8)	4 (33)	4 (31)	4 (22)	5 (42)	7 (44)	32 (30)
**Cytogenetics, n (%)**									
High risk[c]	2 (17)	4 (33)	3 (25)	3 (25)	2 (15)	6 (33)	2 (17)	2 (13)	24 (22)
t(4;14)	1 (8)	1 (8)	0 (0)	1 (8)	1 (8)	2 (11)	1 (8)	1 (6)	8 (7)
t(14;16)	1 (8)	0 (0)	1 (8)	0 (0)	0 (0)	0 (0)	0 (0)	0 (0)	2 (2)
17p13del	1 (8)	3 (25)	2 (17)	2 (17)	1 (8)	4 (22)	1 (8)	1 (6)	15 (14)
1q21+	0 (0)	4 (33)	2 (17)	6 (50)	6 (46)	4 (22)	1 (8)	0 (0)	23 (21)
Del 1p	0 (0)	0 (0)	2 (17)	1 (8)	3 (23)	1 (6)	1 (8)	0 (0)	8 (7)
Other	4 (33)	1 (8)	2 (17)	1 (8)	3 (23)	3 (17)	0 (0)	2 (13)	16 (15)
Missing	2 (17)	1 (8)	3 (25)	2 (17)	2 (15)	1 (6)	1 (8)	2 (13)	14 (13)

**Hypogamma-globulinemia, n (%)**	1 (8)	0 (0)	0 (0)	0 (0)	0 (0)	0 (0)	0 (0)	0 (0)	1 (1)

Percentages may not equate to 100 due to rounding. [a]Defined as the time from first dose to last contact or death; [b]paramedullary disease was not included; [c]if the patient had any of the following cytogenetics: t(4;14), t(14;16), or 17p13del.

ECOG, Eastern Cooperative Oncology Group; mAb, monoclonal antibody; Ig, immunoglobulin; ISS, International Staging System; LOT, line of therapy; MM, multiple myeloma; Q3W, every 3 weeks; Q6W, every 6 weeks; S/D, step-down.
