# Supplementary material for: Efficacy and Safety of Belantamab Mafodotin with Bortezomib plus Dexamethasone in Patients with Relapsed/Refractory Multiple Myeloma: The DREAMM-6 Arm B Trial
Source: Clin Cancer Res. 2026 Mar 2;32(10):1962–72. doi: 10.1158/1078-0432.CCR-25-3216 (PMC13176820; doi:10.1158/1078-0432.CCR-25-3216)
Supplement: Supplementary Table S7 — Belantamab mafodotin dose reductions and dose delays [file ccr-25-3216_supplementary_table_s7_suppts7.pdf]

Supplementary Table S7. Belantamab mafodotin dose reductions and dose delays

Treatment exposure	1.9 mg/kg Q6W n=12	1.9 mg/kg Q3W n=12	2.5–1.9 mg/kg S/D Q6W n=12	2.5 mg/kg Q6W n=12	2.5 mg/kg split Q3W n=13	2.5 mg/kg Q3W n=18	3.4 mg/kg split Q3W n=12	3.4 mg/kg Q3W n=16	All treated N=107
Any dose reduction[b], n (%)	NA	NA	NA	6 (50)	5 (38)	15 (83)	6 (50)	8 (50)	40 (37)
Total reduction events, no.	NA	NA	NA	6	6	15	12	12	51
Reason for reduction[c]									
AE	NA	NA	NA	5 (83)	4 (67)	14 (93)	12 (100)	10 (83)	45 (42)
Other	NA	NA	NA	1 (17)	2 (33)	1 (7)	0	2 (17)	6 (6)
Any dose delay, n (%)	1 (8)	2 (17)	3 (25)	1 (8)	2 (15)	5 (28)	6 (50)	2 (13)	22 (21)
Total delay events, no.	2	5	3	1	2	6	11	2	32

Duration of delay, days, no. of events/total events (%)									
1–21	0/2 (0)	3/5 (60)	1/3 (33)	0/1 (0)	2/2 (100)	4/6 (67)	10/11 (91)	0/2 (0)	20/32 (63)
22–42	2/2 (100)	0/5 (0)	1/3 (33)	0/1 (0)	0/2 (0)	0/6 (0)	1/11 (9)	2/2 (100)	6/32 (19)
>42	0/2 (0)	2/5 (40)	1/3 (33)	1/1 (100)	0/2 (0)	2/6 (33)	0/11 (0)	0/2 (0)	6/32 (19)
Reason for delay[c], no. of events/total events									
AE	2/2 (100)	5/5 (100)	3/3 (100)	1/1 (100)	1/2 (50)	4/6 (67)	10/11 (91)	1/2 (50)	27/32 (84)
Scheduling conflict	0/2 (0)	0/5 (0)	0/3 (0)	0/1 (0)	0/2 (0)	0/6 (0)	1/11 (9)	1/2 (50)	2/32 (6)
Other reason	0/2 (0)	0/5 (0)	0/3 (0)	0/1 (0)	1/2 (50)	2/6 (33)	0/11 (0)	0/2 (0)	3/32 (9)

[a]belantamab mafodotin dose reductions were not allowed for the 1.9 mg/kg cohorts; [b]percentages are calculated based on the total number of events, and patients may have been counted multiple times within the same reason if patients had multiple events for the same reason. Patients in the 3.4 mg/kg cohorts could have a first dose reduction to 2.5 mg/kg, and a subsequent reduction to 1.9 mg/kg, if needed. Patients in split dose cohorts also had the same dose reductions but administered in 2 equal parts on days 1 and 8 of the Cycle. The earliest dose reduction occurred in Cycle 1 in the 3.4

mg/kg Split dose cohort (n=2), with a dose reduction in the 2.5 mg/kg Single cohort occurring at Week 4, and the 2.5 mg/kg Split cohort having a dose reduction in Week 4 Day 8, the 2.5 mg/kg Stretch, and 3.4 mg/kg Single cohorts both had the first dose reduction in Week 7. Patients could continue with the original dose after a dose delay but once a dose was reduced it was not returned to the original dose.

AE, adverse event; NA, not applicable; no., number; Q3W, every 3 weeks; Q6W, every 6 weeks; S/D, step-down.
