# Supplementary material for: Efficacy and Safety of Belantamab Mafodotin with Bortezomib plus Dexamethasone in Patients with Relapsed/Refractory Multiple Myeloma: The DREAMM-6 Arm B Trial
Source: Clin Cancer Res. 2026 Mar 2;32(10):1962–72. doi: 10.1158/1078-0432.CCR-25-3216 (PMC13176820; doi:10.1158/1078-0432.CCR-25-3216)
Supplement: Supplementary Table S8 — Ocular and non-ocular safety summary [file ccr-25-3216_supplementary_table_s8_suppts8.pdf]

Supplementary Table S8. Ocular and non-ocular safety summary

Non-ocular AESI, n (%)	1.9 mg/kg Q6W (n=12)	1.9 mg/kg Q3W (n=12)	2.5–1.9 mg/kg S/D Q6W (n=12)	2.5 mg/kg Q6W (n=12)	2.5 mg/kg split Q3W (n=13)	2.5 mg/kg Q3W (n=18)	3.4 mg/kg split Q3W (n=12)	3.4 mg/kg Q3W (n=16)	All treated (N=107)
Thrombocytopenia or platelet count decreased	9 (75)	12 (100)	10 (83)	11 (92)	11 (85)	13 (72)	12 (100)	14 (88)	92 (86)
Grade 3 or 4	8 (67)	11 (92)	9 (75)	9 (75)	11 (85)	11 (61)	11 (92)	12 (75)	82 (77)
Any infusion-related reactions[a]	0 (0)	2 (17)	1 (8)	2 (17)	2 (15)	3 (17)	3 (25)	1 (6)	14 (13)
**Ocular AESIs**									
Protocol-defined grading, any ocular event, n (%)	10 (83)	11 (92)	12 (100)	12 (100)	10 (77)	18 (100)	10 (83)	16 (100)	99 (93)

Grade ≥2 ocular event (number of events)[b]	16	15	19	20	18	38	14	28	168
Resolved[c], n (%)	10 (63)	7 (47)	7 (37)	14 (70)	12 (67)	24 (63)	11 (79)	20 (71)	105 (63)
Duration of event, days, median (range)	109.5 (50–219)	187.0 (85–430)	118.0 (43–420)	114.5 (22–402)	116.5 (8–713)	145.0 (25–717)	111.0 (22–933)	84.5 (22–1012)	120.0 (8–1012)
Time to first Grade ≥2 ocular event, days, median (range)	30.0 (17.0–62.0)	30.5 (17.0–64.0)	21.5 (17.0–87.0)	22.0 (17.0–82.0)	39.0 (22.0–86.0)	40.5 (21.0–106.0)	41.0 (21.0–49.0)	22.0 (16.0–62.0)	n/c
Grade 3 or 4, n (%)	7 (58)	10 (83)	11 (92)	9 (75)	8 (62)	15 (83)	7 (58)	15 (94)	82 (77)
NCI-CTCAE v4.03 graded, any oAR	9 (75)	12 (100)	12 (100)	12 (100)	10 (77)	18 (100)	10 (83)	16 (100)	99 (93)
Grade 3 or 4	4 (33)	8 (67)	8 (67)	6 (50)	7 (54)	12 (67)	5 (42)	12 (75)	62 (58)

Any oAR related to belantamab mafodotin, n (%)	9 (75)	11 (92)	12 (100)	12 (100)	10 (77)	18 (100)	10 (83)	16 (100)	98 (92)
>20% incidence in any cohort									
Keratopathy	9 (75)	11 (92)	12 (100)	12 (100)	10 (77)	15 (83)	10 (83)	15 (94)	94 (88)
Visual acuity reduced	4 (33)	6 (50)	4 (33)	4 (33)	1 (8)	2 (11)	2 (17)	3 (19)	26 (24)
Vision blurred	1 (8)	3 (25)	5 (42)	5 (42)	5 (38)	12 (67)	3 (25)	7 (44)	41 (38)
Eye pain	0 (0)	2 (17)	0 (0)	1(8)	3 (23)	4 (22)	1 (8)	2 (13)	13 (12)
Dry eye	0 (0)	1 (8)	0 (0)	3 (25)	1 (8)	4 (22)	0 (0)	4 (25)	13 (12)
Photophobia	0 (0)	1 (8)	0 (0)	1 (8)	4 (31)	6 (33)	1 (8)	2 (13)	15 (14)
**Any SAE, n (%)**									
Related to any study treatment	4 (33)	3 (25)	4 (33)	3 (25)	2 (15)	5 (28)	3 (25)	4 (25)	28 (26)
Fatal	1 (8)	0 (0)	1 (8)	1 (8)	0 (0)	1 (6)	1 (8)	2 (13)	7 (7)

Related to study treatment	0 (0)	0 (0)	1 (8)	1 (8)	0 (0)	0 (0)	1 (8)[d]	1 (6)	4 (4)

The protocol-defined scale for ocular events captures both ocular findings and visual acuity changes based on best corrected visual acuity score and slit lap findings. [a]all events were Grade ≤2; [b]where data on event resolution status were provided; [c]by final analysis, to Grade 1 or below; [d]primary cause of death was disease progression with sepsis considered related to belantamab mafodotin, bortezomib and dexamethasone as a secondary cause of death.

AESI, adverse event of special interest; n/c, not calculated; NCI-CTCAE, National Cancer Institute-Common Toxicity Criteria for Adverse Events; Q3W, every 3 weeks; Q6W, every 6 weeks; SAE, serious adverse event; S/D, step-down.
