# Supplementary material for: Efficacy and Safety of Belantamab Mafodotin with Bortezomib plus Dexamethasone in Patients with Relapsed/Refractory Multiple Myeloma: The DREAMM-6 Arm B Trial
Source: Clin Cancer Res. 2026 Mar 2;32(10):1962–72. doi: 10.1158/1078-0432.CCR-25-3216 (PMC13176820; doi:10.1158/1078-0432.CCR-25-3216)
Supplement: Supplementary Table S9 — COVID-19 [file ccr-25-3216_supplementary_table_s9_suppts9.pdf]

Supplementary Table S9. COVID-19

Patients, n (%)	1.9 mg/kg Q6W n=12	1.9 mg/kg Q3W n=12	2.5–1.9 mg/kg S/D Q6W n=12	2.5 mg/kg Q6W n=12	2.5 mg/kg split Q3W n=13	2.5 mg/kg Q3W n=18	3.4 mg/kg split Q3W n=12	3.4 mg/kg Q3W n=16	All treated N=107
**COVID-19**	0 (0)	3 (25)	4 (33)	0 (0)	2 (15)	0 (0)	1 (8)	0 (0)	10 (9)
**Grade 3–4**	0 (0)	1 (8)	1 (8)	0 (0)	0 (0)	0 (0)	0 (0)	0 (0)	2 (2)
**Fatal**	0 (0)	0 (0)	0 (0)	0 (0)	0 (0)	0 (0)	0 (0)	0 (0)	0 (0)
**COVID-19 pneumonia, any grade**	0 (0)	0 (0)	1 (8)	1 (8)	0 (0)	0 (0)	0 (0)	0 (0)	2 (2)
**Grade 3–4**	0 (0)	0 (0)	0 (0)	0 (0)	0 (0)	0 (0)	0 (0)	0 (0)	0 (0)
**Fatal**	0 (0)	0 (0)	1 (8)	1 (8)	0 (0)	0 (0)	0 (0)	0 (0)	2 (2)

Q3W, every 3 weeks; Q6W, every 6 weeks; S/D, step-down.
