# Supplementary material for: Efficacy and Safety of Belantamab Mafodotin with Bortezomib plus Dexamethasone in Patients with Relapsed/Refractory Multiple Myeloma: The DREAMM-6 Arm B Trial
Source: Clin Cancer Res. 2026 Mar 2;32(10):1962–72. doi: 10.1158/1078-0432.CCR-25-3216 (PMC13176820; doi:10.1158/1078-0432.CCR-25-3216)
Supplement: Supplementary Table S10 — Summary of MRD negativity rate for patients with ≥VGPR [file ccr-25-3216_supplementary_table_s10_suppts10.pdf]

## Supplementary Table S10 Summary of MRD negativity rate for patients with ≥VGPR

| | Belantamab mafodotin 1.9 mg/kg | | Belantamab mafodotin 2.5 mg/kg | | | | Belantamab mafodotin 3.4 mg/kg | |
|---|---|---|---|---|---|---|---|---|
| **Cohort** | **Q6W (n=12)** | **Q3W (n=12)** | **2.5–1.9 mg/kg S/D Q6W (n=12)** | **Q6W (n=12)** | **Split Q3W (n=13)** | **Q3W (n=18)** | **Split Q3W (n=12)** | **Q3W (n=16)** |
| Patients with MRD assessment, n | 2 | 2 | 5 | 3 | 3 | 4 | 4 | 3 |
| MRD negativity rate*, n (%) | 2 (17) | 2 (17) | 2 (17) | 1 (8) | 0 | 3 (17) | 2 (17) | 1 (6) |

*Percentage values were based on all patients in the all-treated population.

MRD, minimal residual disease; Q3W, every 3 weeks; Q6W, every 6 weeks; S/D, step-down; VGPR, very good partial response.
