# Supplementary material for: Efficacy and Safety of Belantamab Mafodotin with Bortezomib plus Dexamethasone in Patients with Relapsed/Refractory Multiple Myeloma: The DREAMM-6 Arm B Trial
Source: Clin Cancer Res. 2026 Mar 2;32(10):1962–72. doi: 10.1158/1078-0432.CCR-25-3216 (PMC13176820; doi:10.1158/1078-0432.CCR-25-3216)
Supplement: Supplementary Table S11 — Summary of belantamab mafodotin (ADC) non-compartmental PK parameters at Cycle 1 Day 1, Cycle 1 Day 8 (split cohorts only) and during Cycle 1. [file ccr-25-3216_supplementary_table_s11_suppts11.pdf]

Supplementary Table S11. Summary of belantamab mafodotin (ADC) non-compartmental PK parameters at Cycle 1 Day 1, Cycle 1 Day 8 (split cohorts only) and during Cycle 1.

	Belantamab mafodotin 1.9 mg/kg		Belantamab mafodotin 2.5 mg/kg				Belantamab mafodotin 3.4 mg/kg	
**Cohort**	**Q6W (n=12)**	**Q3W (n=12)**	**2.5–1.9 mg/kg S/D Q6W (n=12)**	**Q6W (n=12)**	**Split Q3W (n=13)**	**Q3W (n=18)**	**Split Q3W (n=12)**	**Q3W (n=16)**
**Cycle 1 Day 1**								
C_{max} (µg/mL)	52.24 (31.9)	49.50 (21.4)	61.16 (36.7)	51.29 (26.0)	21.34 (34.7)	46.95 (23.2)	27.79 (37.3) [n=11]	64.00 (38.6)
t_{max} (h)	1.90 (0.63, 22.50)	1.13 (0.55, 2.62)	2.03 (0.63, 25.95)	1.18 (0.62, 2.57)	1.23 (0.50, 2.22)	1.31 (0.45, 2.70)	0.58 (0.50, 2.20) [n=11]	2.00 (0.52, 3.83)
t_{last} (h)	540 (76, 1052)	506 (504, 1221)	1010 (501, 1489)	755 (23, 2019)	505 (145, 1351)	507 (168, 577)	503 (240, 1585)	506 (2, 2023)
C-EOI (µg/mL)	46.01 (30.1)	44.86 (17.0)	49.77 (45.8)	47.62 (25.7)	20.11 (28.3)	43.18 (22.9)	27.58 (37.9) [n=11]	59.81 (41.0)
**Cycle 1 Day 8**								
C_{max} (µg/mL)	–	–	–	–	24.46 (32.1)	–	31.96 (32.8)	–
t_{max} (h)	–	–	–	–	0.73 (0.52, 336.75)	–	0.64 (0.40, 2.05)	–
C-EOI (µg/mL)	–	–	–	–	21.72 (41.6)	–	31.60 (32.2)	–
**Cycle 1**								
C_{trough} (µg/mL)	1.01 (47.2) [n=4]	1.70 (55.6) [n=11]	0.71 (38.6) [n=4]	0.76 (32.3)	3.51 (41.8) [n=12]	2.30 (55.8) [n=15]	4.78 (53.1)	1.88 (65.7) [n=14]
$AUC_{0\text{-}504}$ (µg·h/mL)	6130 (23.8) [n=7]	4452 (23.3) [n=10]	6074 (37.1) [n=4]	5396 (20.5)	4342 (22.4) [n=7]	5014 (31.9) [n=15]	5702 (26.3) [n=8]	6230 (35.0) [n=11]
$AUC_{0\text{-}1008}$ (µg·h/mL)*	7085 (24.8) [n=5]	–	7396 (25.9) [n=10]	6487 (26.2)	–	–	–	–

Data are presented as geometric mean (%CV) [number of patients evaluated, if different from overall cohort], except t_{max} and t_{last}, which are presented as median (min, max). %CV was calculated as 100*SQRT [exp(SD^2)−1]), where SD represents the SD of the data on a log scale.

*$AUC_{0–1008}$ was derived only for the Q6W and S/D Q6W cohorts.

ADC, antibody–drug conjugate; AUC_{0-x}, area under the concentration–time curve from time 0 to fixed time x; C-EOI, concentration at the end of infusion; C_{max}, maximum plasma concentration; C_{trough}, trough concentration; CV, coefficient of variation; Q3W, every 3 weeks; Q6W, every 6 weeks; S/D, step-down; SD, standard deviation; SQRT, square root; t_{last}, time since first dose of last observed quantifiable concentration; t_{max}, time to maximum plasma concentration
