# Supplementary material for: Efficacy and Safety of Belantamab Mafodotin with Bortezomib plus Dexamethasone in Patients with Relapsed/Refractory Multiple Myeloma: The DREAMM-6 Arm B Trial
Source: Clin Cancer Res. 2026 Mar 2;32(10):1962–72. doi: 10.1158/1078-0432.CCR-25-3216 (PMC13176820; doi:10.1158/1078-0432.CCR-25-3216)
Supplement: Supplementary Table S12 — Summary of patients with ≥12.5-point deterioration from baseline in the OSDI visual related function subscale score [file ccr-25-3216_supplementary_table_s12_suppts12.pdf]

Supplementary Table S12. Summary of patients with ≥12.5-point deterioration from baseline in the OSDI visual related function subscale score

Cohort	Belantamab mafodotin 1.9 mg/kg Q6W (n=12)	Belantamab mafodotin 1.9 mg/kg Q3W (n=12)	Belantamab mafodotin 2.5 mg/kg 2.5–1.9 mg/kg S/D Q6W (n=12)	Belantamab mafodotin 2.5 mg/kg Q6W (n=12)	Belantamab mafodotin 2.5 mg/kg Split Q3W (n=13)	Belantamab mafodotin 2.5 mg/kg Q3W (n=18)	Belantamab mafodotin 3.4 mg/kg Split Q3W (n=12)	Belantamab mafodotin 3.4 mg/kg Q3W (n=16)
**Meaningful within-patient improvement for visual related function subscale score from first meaningful worsening (≥12.5 Improvement) – n (%)**								
Yes	5 (71)	5 (63)	6 (86)	6 (75)	5 (83)	11 (92)	3 (38)	7 (54)
No	2 (29)	3 (38)	1 (14)	2 (25)	1 (17)	1 (8)	5 (63)	6 (46)
**Time to 12.5-point deterioration for visual related function subscale score from baseline (days)**								
n	7	8	7	8	6	12	8	13
Median<br>(Min, Max)	43.0<br>(22, 85)	64.5<br>(23, 190)	51.0<br>(22, 372)	53.5<br>(22, 127)	44.5<br>(22, 87)	47.5<br>(22, 212)	58.0<br>(25, 94)	43.0<br>(22, 86)
**Time to 12.5-point improvement for visual related function subscale score from first meaningful worsening (days)**								
n	5	5	6	6	5	11	3	7
Median<br>(Min, Max)	23.0<br>(22, 64)	64.0<br>(23, 407)	55.0<br>(22, 332)	65.0<br>(22, 478)	64.0<br>(15, 210)	62.0<br>(21, 211)	57.0<br>(22, 449)	65.0<br>(43, 252)

OSDI, Ocular Surface Disease Index; Q3W, every 3 weeks; Q6W, every 6 weeks; S/D, step-down.
